# Financial relationships between patient and consumer representatives and the health industry: A systematic review

**DOI:** 10.1111/hex.13013

**Published:** 2019-12-19

**Authors:** Joanne Khabsa, Aline Semaan, Amena El‐Harakeh, Assem M. Khamis, Serena Obeid, Hussein A. Noureldine, Elie A. Akl

**Affiliations:** ^1^ Clinical Research Institute American University of Beirut Medical Center Beirut Lebanon; ^2^ Center for Research on Population and Health American University of Beirut Beirut Lebanon; ^3^ Center for Systematic Reviews on Health Policy and Systems Research (SPARK) American University of Beirut Beirut Lebanon; ^4^ Faculty of Arts and Sciences American University of Beirut Beirut Lebanon; ^5^ Faculty of Medicine Lebanese American University Byblos Lebanon; ^6^ Department of Internal Medicine American University of Beirut Beirut Lebanon; ^7^ Department of Health Research Methods, Evidence, and Impact (HE&I) McMaster University Hamilton ON Canada

**Keywords:** conflict of interest, consumer, funding, patient representatives, systematic review

## Abstract

**Background:**

Patients and consumers are increasingly engaged in health policymaking, research and drug regulation. Having financial relationships with the health industry creates situations of conflicts of interest (COI) and might compromise their meaningful and unbiased participation.

**Objective:**

To synthesize available evidence on the financial relationships between the health industry and patient and consumer representatives and their organizations.

**Methods:**

We systematically searched MEDLINE and EMBASE. We selected studies and abstracted data in duplicate and independently. We reported on outcomes related to financial relationships of individuals with, and/or funding of organizations by the health industry.

**Results:**

We identified a total of 14 510 unique citations, of which 24 reports of 23 studies were eligible. Three studies (13%) addressed the financial relationship of patient and consumer representatives with the health industry. Of these, two examined the proportion of public speakers in drug regulatory processes who have financial relationships; the proportions in the two studies were 25% and 19% respectively. Twenty studies (87%) addressed funding of patient and consumer organizations. The median proportion of organizations that reported funding from the health industry was 62% (IQR: 34%‐69%) in questionnaire surveys, and 75% (IQR: 58%‐85%) in surveys of their websites. Among organizations for which there was evidence of industry funding, a median proportion of 29% (IQR: 27%‐44%) acknowledged on their websites receiving that funding.

**Conclusion:**

Financial relationships between the health industry and patient and consumer representatives and their organizations are common and may not be disclosed. Stricter regulation on disclosure and management is needed.

## 1 INTRODUCTION

Patients and health consumers have become influential key players in health policy, research and regulation. In the area of policymaking, patients and health consumers can lobby and/or collaborate with policymakers to set priorities, and shape health policies and programmes.^1^, ^2^ In the research area, they are increasingly involved in primary research,^3^ evidence synthesis,^4^ as well as clinical and public health guideline development.^5^ In the regulatory field, patient representatives serve as voting members in United States (US) Food and Drug Administration (FDA) drug advisory committees,^6^ as well as invited speakers.^7^ In Canada, patient groups participate in drug reviews and voice their position on drug funding decisions.^8^ This increased interest in patients’ and health consumers’ engagement is further reflected in the emergence of programmes that support patient and public participation in the above activities such as INVOLVE^9^ and the FDA Patient Representative Program℠.^10^

There is an increasing number of reports about the financial relationship between patient and consumer representatives and their organizations, and the health industry; including pharmaceutical, device and biotechnology industries. A survey of US FDA drug advisory committee meetings found that the proportion of public speakers with conflicts of interest (COI) emanating from the receipt of personal fees or from their organization's financial ties with the industry was 30%.^11^ Similarly, a survey of a national sample of patient advocacy organizations in the United States revealed that 67% of organizations reported receiving industry funding, with around 12% having more than half of their funding generated by the industry.^12^

The financial relationship between the health industry and patient and consumer representatives may compromise their independence and create situations of COI. For example, the position of patients’ organizations on the Centers for Disease Control and Prevention's (CDC)’s guidelines for prescribing opioids for chronic pain was closely associated with receiving funding from the opioid industry.^13^ Similarly, public speakers with COI during meetings of the Anesthetic and Analgesic Drug Products Advisory Committee were more likely to support drug approval than those without COI.^14^ Box 1 describes three other well‐publicized cases illustrating this situation.

Box 1 **Case studies of industry influence on patients’ organizations**

**"The Champion of Painkillers"**

An investigation by ProPublica and the Washington Post revealed that the American Pain Foundation (APF) received 90% of its funding in 2010 from the drug and device industry. The investigation referred to the APF as ‘the champion of painkillers’. In addition, APF’s board members included physicians paid by the pharmaceutical industry. This translated into the engagement of APF in various activities that misled policymakers and the public. These include blame‐shifting to doctor overprescribing, backing up the drug industry in court, asking patients to refute negative stigma associated with painkillers, and publishing outdated materials and guides on the APF’s website that use factual numbers to demote opioids’ harms, while promoting those of other pain relievers.^15^, ^16^ The APF shut down in 2012 following a letter sent by the US Senate Finance Committee enquiring about its financial relationships with opioid makers.^17^

**Lobbying to reimburse but none to reduce prices**

In October 2011, the National Institute for Excellence in Health and Social Services (INESSS) in Quebec recommended the Ministry of Health against the reimbursement of three lung cancer drugs for failing the cost‐effectiveness criterion. A month later, ‘Coalition Priorité Cancer’ (CPC) was successful in lobbying the Minister against the INESSS’ recommendation, raising scepticism about the patient group's affiliation with the industry.^18^ A later investigation by Hughes and Williams‐Jones revealed that the advocacy group received funding from 13 pharmaceutical companies, including the manufacturers of the three drugs initially rejected by INESSS.^19^ Moreover, CPC sought about 60%‐65% of its annual budget from the pharmaceutical industry in 2011.^18^ This was not an isolated occurrence, as CPC has taken the industry's position on multiple occasions, and the majority of its industry‐supported activities deal with the issue of cancer drug reimbursement. CPC’s records show a persistent lobbying of health insurers to reimburse cancer drugs, and no evidence of attempts to urge drug manufacturers to reduce their prices.^18^

**"Percentage of money from pharma has been higher than we have wanted it to be"**

The National Alliance on Mental Illness (NAMI) represents one of the leading and most influential advocacy groups for mental illness in the US. NAMI has received major donations from pharmaceutical companies, with over $6 million a year from the pharmaceutical industry.^20^, ^21^ Although the Alliance has refused for years to disclose specific funding activities, about three quarters ($23 million) of its donations between 2006 and 2008 were from the industry according to investigations by Senator Charles E. Grassley, the Republican of Iowa and New York Times.^22^, ^23^ In addition, NAMI’s executive director indicated that ‘for at least the years of ’07, ’08 and ’09, the percentage of money from pharma has been higher than we have wanted it to be’.^22^ The relationship with the industry has also been shaped as the provision of ‘direct advice about how to advocate forcefully for issues that affect industry profits’. For instance, NAMI was urged to lobby against state efforts to limit access to mental health drugs^22^ such as the FDA’s black‐box warning on antidepressants.^24^

This study aimed to synthesize the available evidence on the financial relationships between the health industry and patient and consumer representatives and their organizations.

## 2 METHODS

### 2.1 Design overview and definitions

We conducted a systematic review based on a protocol registered in the International Prospective Register of Systematic Reviews (PROSPERO).^25^ We referred to the following definitions:

- Health industry: refers to any industry related to health care, including, but not limited to, pharmaceutical, device and biotechnology industries. We included studies that referred to ‘health industry’ without specifying its type(s);
- Financial relationships: refers to either (a) COI of individuals representing patients and consumers with the health industry or (b) the funding of organizations representing patients and consumers by the health industry.

### 2.2 Eligibility criteria

We included studies meeting the following eligibility criteria:

- Study design: we included primary research articles, including surveys and qualitative studies. We excluded case reports, policy briefs, economic studies, technical reports, conference abstracts, editorials, opinion pieces, consensus documents, reviews of the literature, book chapters and books;
- Population: we included studies in which the unit of analysis was individuals or organizations representing patients and consumers. We excluded studies in which the unit of analysis was pharmaceutical companies, and studies that did not report results for the population of interest separately from other populations such as experts and professional organizations;
- Topic: we included studies addressing financial relationships between the health industry and patient and consumer representatives.

### 2.3 Search

We developed with the help of an experienced librarian a search strategy for MEDLINE (1946 to July 2018) and EMBASE (1947 to July 2018) databases. The search strategy combines both keywords and MeSH terms relevant to the concepts of funding and COI, and patients’ representatives (Appendix S1). We used relevant studies identified by a pilot search to refine the definitive search strategy. We did not restrict our search by language, year of publication or study design. In addition, we performed both backward and forward citation tracking (up to March 2019) to identify further eligible studies.

### 2.4 Article selection

Three groups of two reviewers assessed in duplicate and independently the titles and abstracts of citations identified by the search for potential eligibility. We obtained the full text of any citation judged as potentially eligible by at least one reviewer. Subsequently, the reviewers screened in duplicate and independently the full texts using a standardized screening form. They resolved their disagreements by discussion or with the help of a third reviewer as needed. We recorded reasons for exclusion and summarized the results of the selection process using a PRISMA flow diagram. All reviewers completed calibration exercises before starting the screening process.

### 2.5 Data extraction

We developed and pilot‐tested a standardized data extraction form with detailed instructions. The reviewers completed a calibration exercise and then extracted data in duplicate and independently. They resolved disagreements through discussion or with the help of a third reviewer as needed.

We extracted information about study general characteristics, funding outcomes (e.g. proportion of organizations receiving funding, proportion of organizations acknowledging funding, funding amount and proportional financial contribution), and/or outcomes related to financial relationships of individuals (e.g. disclosed and undisclosed COI).

### 2.6 Data analysis/Synthesis of the results

We summarized the findings of the included studies using evidence synthesis approaches for both quantitative and qualitative data. Also, when possible, we summarized the findings in tabular formats. In our analysis, we considered patients and consumers as one group, even when the included study distinguished between the two.^26^ Similarly, we did not distinguish between advocacy groups and consumer organizations even when included studies did so.^27^

## 3 RESULTS

### 3.1 Study selection and characteristics

Our search of the electronic databases yielded a total of 14 497 unique citations. Additional searches identified 13 additional references. We excluded 186 studies at the full‐text screening stage for the following reasons: not in the health field (n = 2), not about funding or financial relationships of individuals (n = 49), not about patients’ representatives or patients’ organizations (n = 40), not of the study design of interest (n = 73), not about the health industry (n = 12), not outcomes of interest (n = 4) or other reasons (n = 6; Appendices S2 and S3).

**Figure 1:**
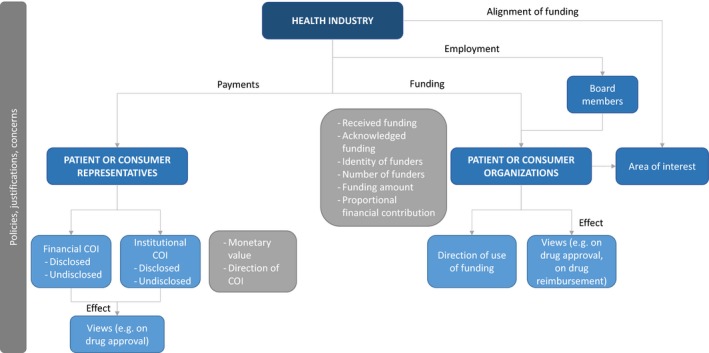
Outcomes relating to patient and consumer representatives and their organizations

We included a total of 24 papers reporting on 23 studies; two papers reported on the same study^28^, ^29^ (Appendix S2). Of these, three were mixed methods studies,^30^, ^31^, ^32^ one was a qualitative study,^33^ and the rest (n = 19) were quantitative studies. Three studies (13%) addressed patients’ representatives, while 20 (87%) addressed patients’ organizations. We report below findings on patients’ representatives and patients’ organizations, respectively. Figure 1 illustrates the outcomes we report on and how they relate.

### 3.2 Patients’ representatives

We included three eligible studies with patients’ representatives as the population of interest. Table 1 shows the general characteristics of these studies, while Table 2 shows their detailed findings. All studies were about patients’ representatives acting as public speakers at the US FDA drug advisory committee meetings. Two studies included meetings that focused on a specific specialty; respectively, anaesthetic and analgesic drugs,^14^ and oncologic drugs.^11^ The third study included meetings that focused on different specialties.^26^ The three studies included meetings since 2009.

**Table 1:** Characteristics of included studies addressing patients’ representatives (N = 3)

Author Year	Research method	Population and country	Data collected and source	Outcome	Funding of the study	COI of study authors
Abola 2016^11^	Quantitative	Population: 103 public speakers (patients with cancer or representatives of patient advocacy organizations) at 28 FDA^a^ ODAC^b^ meetings (2009‐2014) Country: US	Disclosed COI: review of published meeting transcripts on the FDA^a^ website (2015) Undisclosed COI: online research for whether organization had received funding	Disclosed COI Undisclosed COI	Not reported	None
McCoy 2018^14^	Quantitative	Population: 112 speaking appearances by 91 ‘patients and advocates’ at 15 FDA^a^ AADPAC^c^ meetings related to drug approval (2009‐2017) Country: US	Disclosed COI: review of published meeting transcripts on the FDA^a^ website (2017) Undisclosed COI: Google search for whether organization had received funding (2017)	Disclosed COI Undisclosed COI	Not reported	1/6 member of the AADPAC^c^ committee; 1/6 spouse employment by a patient advocacy organization
Graham 2016^26^	Quantitative	Population: 315 participations by patient representatives and consumer representatives at 167 FDA^a^ drug advisory committee meetings (2009‐2012) Country: US	Disclosed COI: review of online FDA^a^ archives Undisclosed COI: online research into representatives’ backgrounds and employment histories	Total COI (disclosed and undisclosed)	University of Wisconsin–Milwaukee Office of Research and College of Letters & Science	4/6 reported financial relationships

a Food and Drug Administration.

b Oncologic Drugs Advisory Committee.

c Anesthetic and Analgesic Drug Products Advisory Committee.

**Table 2:** Results of studies addressing COI of patient representatives (N = 3)

Author Year	Disclosed COI	Undisclosed COI	Disclosed and undisclosed COI	Additional results
Abola 2016^11^	Total financial: 30.1% (31/103) o Personal (travel fees): 10.7%^a^ (11/103) o Organizational: 17.5%^a^ (18/103) o Personal (travel fees) and organizational: 1.9%^a^ (2/103) Investigators in seminal trials: 1.9% (2/103) Organizational but not financial: 1.9%^a^ (2/103)	Financial (organizational): 1.9%^b^ (2/103)	Not reported	None of the speakers who did not support marketing approval reported a financial association with a sponsoring company
McCoy 2018^14^	Total: 19.6% (22/112) Travel fees and other meeting expenses: 15.2% (17/112) Organizational: 4.5% (5/112) Investigator on drug trial: 0.9% (1/112)	Financial (organizational): 18.8% (21/112) Prior to the meeting in question: 5.4% (6/112) Of indeterminate date: 13.4% (15/112)	Total (prior to the meeting in question): 25% (28/112)	Speakers who disclosed a conflict of interest were at higher odds of supporting drug approval than those who did not (OR = 6.07; 95% CI: 1.29‐28.56; *P*‐value = .02). Findings were similar when undisclosed financial associations were included in the analysis
Graham 2016^26^	Not reported separately	Not reported separately	Total: 19% (61/315) Personal: 3.2%^a^ (10/315) ‘Imputed^c^’: 13.33%^a^ (42/315) ‘Personal and imputed^c^’: 2.9%^a^ (9/315)	Direction of COI: COI involving manufacturer of the principal drug discussed: 11.5%^a^ (7/61) COI involving direct competitor: 45.9%^a^ (28/61) COI involving marketplace competitor: 42.6%^a^ (26/61) Monetary value: ≥ $50 000:57.4%^a^ (35/61) <$50 000:1.6%^a^ (1/61) Not disclosed: 40.9%^a^ (25/61)

a Value calculated by the authors.

b Speakers had disclosed organizational but not financial COI and subsequent searches of online information showed that the specified organizations had received financial support from the sponsor before the meeting.

c COI resulting from a relationship between the industry and a patient advocacy organization or other types of relationships.

In McCoy et al, the proportion of speaking appearances in which COI was disclosed was around 20%, and the proportion of appearances in which COI was undisclosed was 5%. The proportion of appearances with disclosed and undisclosed COI was 25%.^14^ In Abola and Prasad, the proportion of public speakers who disclosed financial COI was 30%, and the proportion of public speakers who did not disclose that the organizations they represented received financial support from the drug sponsor before the meeting was 2%.^11^ Graham et al found that 19% of participations of public speakers had COI.^26^ The same study found that the monetary value of the conflict was ≥$50 000 for 57% of the participations.^26^ Two studies suggested a positive association between COI and position on drug approval.^11^, ^14^

### 3.3 Patients’ organizations

We included 20 eligible studies evaluating patients’ organizations. Table 3 shows a summary of the general characteristics of these studies, while Appendix S4 provides the detailed general characteristics of each study. The median number of organizations included in each study was 69 (IQR: 53‐157). Studies mostly addressed European (40%) and North American (30%) organizations. Few studies focused on a specific health condition (35%). The most frequent source of data was websites of the organizations (60%). Appendix S5 provides detailed results of each study.

**Table 3:** General characteristics of included studies evaluating patients’ organizations (N = 20)

Study characteristics	n (%)
Number of organizations^a^ (median, IQR)	69 (53‐157)
Country of organization	
Europe	8 (40)
North America	6 (30)
Australia	3 (15)
International^b^	3 (15)
Focused on specific health condition^c^	7 (35)
Study included only pharma sponsored organizations	6 (30)
Focus of the study^d^	
Pharmaceutical industry	19 (95)
Health devices/biotechnology industry	6 (30)
Other or unspecified health industry	3 (15)
Source of data^d^	
Websites of organizations	12 (60)
Questionnaire survey	8 (40)
Annual reports of organizations	7 (35)
Contact of patients’ organizations	4 (20)
Interview	4 (20)
Federal tax forms 990	3 (15)
Submissions in the setting of a regulatory process	2 (10)
Other^e^	4 (20)
Outcome assessed^d^	
Receiving industry funding	13 (65)
Policy for dealing with industry	11 (55)
Reporting on funding amount	9 (45)
Reported funding amount	7 (35)
Reporting on direction of use of funding	7 (35)
Reported direction of use of funding	7 (35)
Reported proportional financial contribution	6 (30)
Reporting on proportional financial contribution	5 (25)
Number of funders	5^f^ (25)
Unrestricted funding	5 (25)
Acknowledging industry funding	5 (25)
Perspectives of stakeholders	4 (20)
Reporting on the identity of funders	3 (15)
Board members with COI	3 (15)
Alignment between funding and industry's interests	2 (10)
Effect of funding	2 (10)
Other^g^	5 (25)

a Median calculated only for included quantitative studies or quantitative parts of mixed methods studies.

b National or international organizations based in the USA, UK, Australia, Canada and South Africa (n = 1); organizations from Scandinavian and English speaking countries (Australia, Canada, Denmark, New Zealand, Norway, Sweden, the United Kingdom and the United States) (n = 1); Poland and Canada (n = 1).

c Cancer (n = 3), rare diseases (n = 1), dermatology (n = 1), paediatric orthopedics (n = 1), 10 major health conditions (cancer, heart disease, diabetes, asthma, cystic fibrosis, epilepsy, depression, Parkinson's disease, osteoporosis, rheumatoid arthritis) (n = 1).

d More than one answer applies.

e Analyses of documentation published by and about the organizations, conferences and seminars hosted by the organizations (ethnographic research) (n = 1); LinkedIn search (n = 1); survey of 2013‐2016 Medicines Australia reports (n = 1); survey of ABPI’s Disclosure UK database, survey of websites of pharmaceutical companies (n = 1).

f Includes ‘number of sponsorships per organization’ (n = 1).

g More than one answer applies: justification for the relationship (n = 4), categorization of the relationship (n = 2), COI concerns (n = 1).

#### 3.3.1 Receiving industry funding

The most frequently reported on outcome was the proportion of organizations that receive industry funding (65%). Six studies were questionnaire surveys.^12^, ^28^, ^30^, ^31^, ^34^, ^35^ The median proportion of organizations that receive industry funding in five studies was 62% (IQR: 34%‐69%). The sixth study reported that ‘more than 80%’ of organizations did not receive funding from the pharmaceutical industry.^34^ Five studies were surveys of organizations’ websites^27^, ^36^, ^37^, ^38^, ^39^ and had a median proportion of organizations receiving industry funding of 75% (IQR: 58%‐85%). Two studies examined the proportion of organizations that formally declared receiving funding in the setting of a regulatory process. In one study, disclosed funding was 87%.^40^ In the other study, total funding (disclosed and undisclosed) was 72%, of which 41% was undisclosed.^41^

#### 3.3.2 Acknowledging industry funding

Five studies (25%) assessed the proportion of organizations for which there was evidence of industry funding and that acknowledged receiving that funding on their websites.^29^, ^32^, ^42^, ^43^, ^44^ In one study, funding information was provided by members of the organizations themselves,^29^ while in the remaining four, funding information was provided by pharmaceutical companies. The median proportion of organizations receiving industry funding and acknowledging it was 29% (IQR: 27%‐44%).

#### 3.3.3 Reporting on the identity and number of industry funders

Three studies assessed reporting on donor identity.^32^, ^36^, ^44^ Two studies found that out of organizations that acknowledged receiving industry funding on their website, 99%^44^ and 34%,^32^ respectively, named donors. The third study found that 67% of sampled organizations disclosed the names of individual donors in their annual reports or on their websites. Two studies compared the donor identities acknowledged on the websites of patients’ organizations with those listed in reports from the pharmaceutical industry.^42^, ^44^ The studies found that, respectively, 25% and 24% of the sponsorships disclosed by patients’ organizations corresponded exactly to the reports made by the industry.

Four studies reported on the number of funders,^37^, ^38^, ^41^, ^42^ with the median number of funders ranging between two 41 and seven,^37^ and the maximum number of funders being 38.^38^ One study found that the median number of sponsorships per organization was three.^45^

#### 3.3.4 Reporting on funding amount

Nine studies (45%) assessed whether organizations reported (yes/no) on the funding amount on their website,^32^, ^36^, ^38^, ^39^, ^42^, ^43^, ^44^ or in surveys.^12^, ^31^ The percentage of organizations reporting on funding amount varied from 0% to 50%.

#### 3.3.5 Reported funding amount received

Seven studies (35%) reported on the exact amount of funding received,^12^, ^31^, ^36^, ^39^, ^40^, ^41^, ^45^ but reported results using different statistics (mean, median and/or range). Three studies used the same cut‐off of $1 000 000: one study using questionnaire surveys found that 9% of organizations received ≥$1 000 000 in a year from for‐profit companies^12^; the remaining two, respectively, found that 25% 36 and 39% 39 of organizations declared on their website receiving ≥$1 000 000 in a year from the drug, device or biotechnology industry.

#### 3.3.6 Reporting on proportional financial contribution

Five studies (25%) assessed whether organizations reported (yes/no) on industry's proportional financial contribution.^32^, ^38^, ^40^, ^42^, ^44^ Four of these studies assessed the percentage of organizations reporting on their websites on the proportion of industry contribution to total income (0%, 0%, 4% and 6%). The fifth study assessed reporting during a regulatory process on industry's proportional contribution to annual budget and found that 20 out of 324 submissions of recommendations about whether drug plans should list drugs for specific indications stated the proportion annual budget attributable to industry donations.^40^

#### 3.3.7 Reported proportional financial contribution received

Six studies (30%) reported on the exact value of the industry's proportional contribution.^12^, ^31^, ^36^, ^39^, ^40^, ^41^ It was not feasible to summarize the findings as the six studies varied very widely in terms of the denominator used: total income (n = 2),^39^, ^41^ budget (n = 2),^31^, ^40^ total donations (n = 1),^36^ any for‐profit industry contribution to total funding and health industry contribution to industry support (n = 1).^12^ They also varied in terms of the statistics (range, median, IQR) and cut‐offs used to report the findings. The authors’ judgement is that the proportional financial contribution of the industry to the organizations’ finances is non‐negligible at the least.

#### 3.3.8 Reporting on direction of use of funding

Seven studies (35%) assessed whether organizations reported (yes/no) on the direction of use of funding.^32^, ^36^, ^38^, ^39^, ^40^, ^42^, ^44^ The median proportion of organizations reporting on the direction of use of funding on their website across six studies was 22% (IQR: 7%‐53%). The one study that assessed disclosures in regulatory settings, found that 82% of declarations naming the donor companies did not specify how the received money was used.^40^

#### 3.3.9 Reported direction of use of funding

Seven studies (35%) reported on the actual direction of use of funding.^27^, ^32^, ^38^, ^40^, ^42^, ^44^, ^45^ The latter varied across studies, but the most common mentioned uses were core operation support, research and educational activities. Three studies, respectively, found that the proportion of organizations receiving unrestricted funding was 10%,^45^ 13%^38^ and 20%.^42^ Two other studies referred to unrestricted funding but without reporting on proportions.^32^, ^44^

#### 3.3.10 Alignment between funding and industry's interests

94% of Eli Lilly's grants to patients’ organizations went to organizations covering the three therapeutic areas that constitute 87% of Eli Lilly's total US sales in 2007.^43^ Fabbri et al found that the main funders of the five consumer groups that received the most funding over the period of 2013‐2016 in Australia were companies that manufactured drugs for conditions covered by these groups and that were under review for subsidization from the government.^45^

#### 3.3.11 Effect of funding

One study suggested an association between funding of patients’ organizations by the pharmaceutical industry and presentation of information about breast cancer screening on the organizations’ websites. The study found that all organizations that accepted support presented biased information, while those that did not, questioned the value of screening.^27^ Another study found no association between funding of patients’ organizations by the pharmaceutical industry and their views about funding of drugs by provincial governments in Canada.^40^

#### 3.3.12 Policy for dealing with industry

Seven studies (35%) assessed the proportion of organizations with a policy for dealing with the industry.^12^, ^32^, ^35^, ^39^, ^41^, ^42^, ^44^ The median proportion was 26% (IQR: 9%‐52%). A mixed methods study found that only three of 13 interviewed organizations had, or were in the process of developing their own codes for interacting with the industry.^31^ The median proportion of organizations specifically stating that they do not accept funding from the industry was 3% across four studies (IQR 1%‐10%).^27^, ^37^, ^39^, ^41^ Another study found that ‘a small number of organizations’ have policies of refusing pharmaceutical industry funding.^30^

#### 3.3.13 Organizations’ board members with financial relationship with the industry

Three studies (15%) surveyed websites of organizations for the COI of their board members. The first study found that 40% of members were current or former drug, device or biotechnology industry executives,^39^ while the second study found that 64% of board members were current or former employees of pharmaceutical companies.^44^ A third study found that the percentage of board members who were current or former industry executives was 13%. However, a LinkedIn search showed a percentage of 54%.^36^

#### 3.3.14 Perspectives of stakeholders

Leto di Priolo et al surveyed a convenience sample of 161 policymakers, cancer patient group representatives and representatives of health professional groups from 12 European countries about their opinion on the relationships between cancer patients’ organizations and the pharmaceutical industry. While the relationship was generally viewed as positive, concerns were raised about the relationship being unequal, conflicts between the industry's interests and advocacy interests, motives behind the funding and the risks to patients’ organizations’ credibility.^46^ Interviews of patients’ organizations by Hemminki et al revealed additional concerns, such as issues of sustainability, conditions imposed by the industry and power balance.^31^ Patient groups interviewed by Jones were also concerned about industry links compromising their credibility, and accusations of promoting drug makers’ products.^32^

Policy actors interviewed by Jones stressed on the importance of disclosure and transparency around industry's relationships with patients’ organizations.^32^ Around 40% of interviewed stakeholder groups by Leto di Priolo et al expressed dissatisfaction with the current management of the relationship between patients’ organizations and the industry.^46^ Respondents had the most positive views about project funding from multiple companies, while core funding was viewed as a negative approach.^46^ Patients’ organizations interviewed by Jones also argued that policies can be developed to manage relationships and ensure transparency. Suggested actions included public disclosure, caps on funding amount, multi‐source funding, editorial independence and the restriction from promotion of industry products.^32^

Influence of the pharmaceutical industry on patients’ organizations was also recognized as a threat to health technology assessments (HTA) by members of appraisal committees in Poland and Canada. The threat could be internal to the HTA process, by having members of these organizations serve on committees. The threat could also be external, by incorporating potentially biased information obtained from these groups.^33^

#### 3.3.15 Other findings

One study categorized the relationships between patients’ organizations and the pharmaceutical industry as corporatist, cautious co‐operation or confrontational.^30^ Another study categorized patients’ organizations as refusers, accepters and non‐disclosers.^32^ Box 2 summarizes the main justifications for the co‐operation of patients’ organizations with the industry reported in four studies.^30^, ^31^, ^32^, ^46^

Box 2 **Justifications for patient organizations’ co‐operation with the industry**

- Communication and transmittal of accurate information between producers and health consumers^31^, ^32^, ^46^
- Low resources (e.g. need for resources for provision of services that benefit patients,^32^ insufficient public funding^30^)
- Common interests
  - Development of new,^46^ safe and effective therapies^30^
  - Drug approval^30^
  - Drug access (e.g. quick access,^46^ better reimbursement of drugs^31^)
  - Promoting adherence to treatments^46^
  - Pharmacovigilance^46^
  - Policymaking^46^
  - Maintaining credibility and trust with other stakeholders^46^
- Learning about good marketing skills^31^
- No source is conflict‐free^32^
- No other choice^30^

## 4 DISCUSSION

### 4.1 Summary of findings

We systematically reviewed the literature for financial associations between the health industry, and patient and consumer representatives and their organizations. A considerable proportion of patients’ representatives acting as public speakers in regulatory processes have financial relationships with the health industry. Similarly, the majority of patients’ organizations receive funding from the industry. The amounts received were non‐negligible, both in absolute numbers, and as a proportion of budget, income or donations. On the other hand, only a minority of these organizations acknowledge funding or have in place a policy regulating these relationships. Funding is unrestricted in a minority of cases and is typically closely aligned with funders’ interests. Studies suggested a positive association between financial relationships and position on drug approval for public speakers. The effect of funding on the views of patients’ organizations remains unclear. We identified various justifications for and concerns about these relationships.

It is important to note that studies on the funding patients’ organizations reported highly variable results. This could be due to the fact that some studies addressed only organizations funded by the industry while others addressed any organization (whether funded or not). The former studies are more likely to find higher average amounts of funding. Similarly, some studies included only organizations with high annual revenue (e.g. >$7.5 million)^39^ while others did not apply such restrictions. Also, in this case, the former studies are more likely to find higher average amounts of funding.

The heterogeneity in the findings could also be explained by the different types of organizations addressed across studies. One would expect substantive variation in the results between small grassroots organizations and larger (more business oriented) organizations. Unfortunately, the studies did not consistently report on the types of the included consumer organizations. In addition, there is no widely agreed upon way to categorize those organizations.

### 4.2 Strengths and limitations

This study has a number of strengths. To our knowledge, this is the first study to systematically review the literature for financial relationships between the health industry and patient and consumer representatives. We followed the Cochrane Collaboration methodology for conducting systematic reviews,^47^ including a comprehensive search strategy, and duplicate and independent selection and data abstraction processes.

The limitations of this study do not relate to its methodology but to the included studies. First, these studies were mostly conducted in high‐income countries which limits the generalizability of their findings to low‐ and middle‐income countries (LMICs). Second, the studies were heterogeneous in terms of design and outcomes reported, which prevented us from calculating a summary estimate in multiple instances. Third, we identified no study in the specific settings of research conduct or guideline development.

### 4.3 Comparison to similar research

Our findings are consistent with those of studies examining the interaction between consumer representatives and other types of industries, namely the tobacco, alcohol, and food and beverage industries.^48^, ^49^, ^50^ These other (non‐health) industries created grassroots organizations that claim to represent the public, while acting as front groups for them—a phenomenon known as ‘astroturfing’.^51^ These front groups engage in opposing legislations and tax reforms, and scare and disease mongering.^48^, ^49^, ^52^ Interaction with health consumers was shown to sit within the industry's larger plan to interfere through different means with all health actors, such as funding agencies, experts, professional organizations, regulatory agencies and health practitioners.^53^

Ozieranski et al. addressed our review question but based on the analysis of industry money going to organizations. They found that 69% of industry money dedicated to patient organizations covering 30 condition areas went to organizations covering five conditions. The authors noted that the funding by donors in these five condition areas was aligned with recent launches of multiple related expensive drugs by these donors.^54^ A US Senate report of opioid industry funding to patients’ organizations also shows that amounts of funding received are high (payments from five opioid manufacturers to 14 selected groups in 2012‐2017 totalled around $9 million), that most grants are restricted, and that payments are also made to individuals such as organizations’ board members.^55^

Considering that there was a considerable proportion of undisclosed financial relationships among patients’ representatives and undisclosed funding among patients’ organizations, we believe that our findings are underestimations of the real prevalence of these financial relationships. In the US, disclosure of financial conflicts is ‘encouraged’ for public participants in FDA advisory committee meetings, but not required.^56^ A comparative analysis of relevant codes of practice and regulation found that the US, compared to the other industrialized nations (i.e. the United Kingdom, Germany, France, Australia and Canada), ‘lacks rigorous principles guiding pharmaceutical company interactions with patient‐advocacy organizations’, including disclosure policies.^24^

On the other hand, there are no public databases in the United States in which payments by the industry to patients’ organizations can be disclosed. Kaiser Health News (KHN) established its ‘Pre$cription For Power' database to track transactions from the pharmaceutical industry to US patient groups.^57^ ‘Disclosure UK’ and ‘La base de données publique Transparence‐Santé’ are databases established in the UK and France, respectively, to track payments between drugs makers and patients’ groups.^24^

### 4.4 Implications for practice

Addressing the engagement of patients’ representatives and their organizations in health policy, research and regulation should further consider this group's interaction with the health industry and the resulting COI. Advocacy efforts should be geared towards expanding funding from sources other than the health industry, such as public funding. The need to support patient and consumer organizations through public funding is highlighted in the literature.^58^, ^59^ A rigorous process of disclosure of relationships should be put in place, along with management policies and vetting for undisclosed links.

Stein et al. described the development of guidelines on collaboration between rare disease patients’ organizations and the biopharmaceutical industry by an expert panel comprised of leaders from the two groups.^60^ Recommendations include seeking funding from multiple companies, a board of directors free from companies’ representatives, and refusing funding for product promotional activities. Ideally, funding should be unrestricted or for a specific activity initiated by the organization. Funding sources should be made transparent by the organization and all benefits should be documented.^60^ While the Sunshine Act provisions of the Affordable Care Act in the US regarding industry's disclosure of payments to physicians and teaching hospitals^61^ do not cover patients or their organizations, there is a case for their expansion to include patients’ organizations, as previously highlighted by McCoy^62^ and Karas et al.^24^

### 4.5 Implications for future research

Future studies should assess the funding of patients’ organizations, and the types and disclosure of financial relationships among patients’ representatives in the research and guideline development settings, and in the setting of LMICs. It would also be interesting to explore whether industry funding varies by whether the consumer organizations can access public funding. Further studies should also assess the uncertainties around the effect of these relationships on the participation of this group in health‐care research and practice. Finally, there is a need to develop frameworks and guidance for disclosure of financial relationships and management when engaging patients and consumers in research, health policy and regulation, or guideline development.

## CONFLICT OF INTEREST

All authors have completed the ICMJE uniform disclosure form at http://www.icmje.org/coi_disclosure.pdf. EAA declares conducting research on the topic of conflict of interest. All other authors declare no conflicts of interest.

## AUTHORS’ CONTRIBUTIONS

EAA conceived the study; EAA and JK designed the study. JK coordinated the study. EAA had full access to all of the data in the study and takes responsibility for the integrity of the data and the accuracy of the data analysis. JK ran the search. JK, AS, AK, AEH, SO and HAN contributed to the study selection process. JK, AS, AEH and SO extracted the data. JK and EAA analysed and interpreted the data. JK wrote the first draft of the manuscript with EAA. All authors critically revised the manuscript and approved the final manuscript. EAA affirms that this manuscript is an honest, accurate and transparent account of the study being reported; that no important aspects of the study have been omitted; and that any discrepancies from the study as planned have been explained.

## ETHICAL APPROVAL

The study involves no human subjects and requires no ethical approval.

## Data Availability

The data sets used and/or analysed during the current study are available from the corresponding author on request.
