# Supplementary material for: Financial relationships between patient and consumer representatives and the health industry: A systematic review
Source: Health Expect. 2019 Dec 19;23(2):483–95. doi: 10.1111/hex.13013 (PMC7104632; doi:10.1111/hex.13013)
Supplement: Supplementary file 1 [file HEX-23-483-s001.docx]

**Appendix 1: Search strategy**

**Database: Ovid MEDLINE(R) and Epub Ahead of Print, In-Process & Other Non-Indexed Citations and Daily <1946 to July 20, 2018>**

1 "Conflict of Interest"/ (9098)

2 ((competing or conflict* or declar*) adj3 (interest? or influence? or relationship?)).mp. [mp=title, abstract, original title, name of substance word, subject heading word, floating sub-heading word, keyword heading word, protocol supplementary concept word, rare disease supplementary concept word, unique identifier, synonyms] (18214)

3 financial support/ or research support as topic/ (25400)

4 ((financial or financing or monetary or industr* or pharmaceutical*) adj3 (pay* or paid or support or contribution? or compensation? or sponsor* or back or backing or (kick adj back*) or incentive? or re?imburse* or subsidi* or award* or endow* or disclos* or tie? or link* or association? or affiliation? or relationship?)).mp. [mp=title, abstract, original title, name of substance word, subject heading word, floating sub-heading word, keyword heading word, protocol supplementary concept word, rare disease supplementary concept word, unique identifier, synonyms] (24959)

5 Disclosure/ (12465)

6 Gift Giving/ (1488)

7 ((gift or gifts or gift-giving) adj3 (disclos* or report* or declar* or reveal* or receiv* or giv* or gave or accept* or award* or admit*)).mp. [mp=title, abstract, original title, name of substance word, subject heading word, floating sub-heading word, keyword heading word, protocol supplementary concept word, rare disease supplementary concept word, unique identifier, synonyms] (1986)

8 (funding or funded or funds).mp. [mp=title, abstract, original title, name of substance word, subject heading word, floating sub-heading word, keyword heading word, protocol supplementary concept word, rare disease supplementary concept word, unique identifier, synonyms] (75322)

9 1 or 2 or 3 or 4 or 5 or 6 or 7 or 8 (139894)

10 Patient Advocacy/ (23232)

11 Consumer Advocacy/ (3250)

12 ((patient? or client? or consumer? or user? or public) adj3 (representative? or delegate? or advoc* or organi?ation? or voice? or response? or advisor? or speaker? or group? or lobby*)).mp. [mp=title, abstract, original title, name of substance word, subject heading word, floating sub-heading word, keyword heading word, protocol supplementary concept word, rare disease supplementary concept word, unique identifier, synonyms] (469090)

13 Caregivers/ (30453)

14 (caregiver? or (care adj giver?) or carer? or surrogate?).mp. [mp=title, abstract, original title, name of substance word, subject heading word, floating sub-heading word, keyword heading word, protocol supplementary concept word, rare disease supplementary concept word, unique identifier, synonyms] (120940)

15 10 or 11 or 12 or 13 or 14 (585464)

16 9 and 15 (8061)

***************************

**Database: Embase <1947 to 2018 July 18>**

#14 #7 AND #13 12036

#13 #8 OR #9 OR #10 OR #11 OR #12 926068

#12 caregiver* OR 'care giver*' OR carer OR carers OR surrogate OR surrogates 175272

#11 'caregiver' 77619

#10 (patient* OR client* OR consumer* OR user* OR public) NEAR/3 (representative* OR delegate* OR advoc* OR organization* OR organisation* OR voice* OR response* OR advisor* OR speaker* OR group* OR lobby*) 760022

#9 'consumer advocacy' 3156

#8 'patient advocacy' 21397

#7 #1 OR #2 OR #3 OR #4 OR #5 OR #6 197121

#6 (gift OR gifts OR 'gift giving') NEAR/3 (disclos* OR report* OR declar* OR reveal* OR receiv* OR giv* OR gave OR accept* OR award* OR admit*) 1715

#5 'gift giving/' 1151

#4 (financial OR financing OR monetary OR industr* OR pharmaceutical*) NEAR/3 (pay* OR paid OR support OR contribution* OR compensation* OR sponsor* OR back OR backing OR kick*back* OR incentive* OR reimburse* OR 're-imburse*' OR subsidi* OR award* OR endow* OR disclos* OR tie* OR link* OR association* OR affiliation* OR relationship*) 63765

#3 'funding'/exp OR 'research support' OR 'funding' OR 'funded' OR 'funds' 116873

#2 (competing OR conflict* OR declar*) NEAR/3 (interest* OR influence* OR relationship*) 22179

#1 'conflict of interest'/exp 10105
