# Supplementary material for: Financial relationships between patient and consumer representatives and the health industry: A systematic review
Source: Health Expect. 2019 Dec 19;23(2):483–95. doi: 10.1111/hex.13013 (PMC7104632; doi:10.1111/hex.13013)
Supplement: Supplementary file 2 [file HEX-23-483-s002.doc]

**Appendix 2: PRISMA flow diagram**

**Screening**

**Included**

**Eligibility**

**Identification**

Records identified through database searching
(n = 20,097)

)

Additional records identified through other sources
(n = 13)

Records after duplicates removed
(n = 14,510)

Records screened
(n = 14,510)

Records excluded
(n = 14,300)

Full-text articles assessed for eligibility
(n = 210)

Studies included in qualitative synthesis
(n = 24 reports of 23 studies)

Full-text articles excluded (n= 186)

- Not health field (n=2)
- Not about funding or financial relationships of individuals (n= 49)
- Not about patient or consumer representatives (n=40)
- Not study design of interest (n= 73)
- Not health industry (n=12)
- Not outcome of interest (n=4)
- Other* (n=6)

*Full text not found (n=3), retracted article (n=1), results not stratified by population group (n=2)
