# Supplementary material for: Financial relationships between patient and consumer representatives and the health industry: A systematic review
Source: Health Expect. 2019 Dec 19;23(2):483–95. doi: 10.1111/hex.13013 (PMC7104632; doi:10.1111/hex.13013)
Supplement: Supplementary file 3 [file HEX-23-483-s003.docx]

**Appendix 3: List of excluded studies**

| ***Not health field*** | ***Not about funding or financial relationships of individuals*** | ***Not about patient or consumer representatives*** | ***Not study design of interest*** | ***Not health industry*** | ***Not outcome of interest*** | ***Other*** |
| --- | --- | --- | --- | --- | --- | --- |
| Van de Bovenkamp, 2011^173^  Williamson, 2016^185^ | Anderson, 2003^6^  Anonymous, 1999b^10^  Anonymous, 2005b^13^  Anonymous, 2007^15^  Antarsh, 1992^19^  Armes, 2009^23^  Baggott, 2018^29^  Barohn, 2016^32^  Bramstedt, 2003^38^  Cahill, 1994^41^  Carleton, 2001^42^  Carroll-Johnson, 2003^43^  Charters, 1993^45^  Cheson, 2010^46^  Clements, 2003^47^  Conter, 2009^54^  Cukor, 2016^56^  Dang, 2016^57^  Dellasega, 2003^61^  Di Pietro, 2012^62^  E. A. Miller, 2015^63^  Entwistle, 2003^67^  Fentiman, 2003^69^  Gillam, 2016^75^  Gillen, 2009^76^  Hale, 1998^83^  Hays, 2016^87^  Hofmann, 2011^93^  J. A. Wagner, 2011^96^  Jaklevic, 2004^97^  Kapp, 1999^100^  M. P. Adams, 2014^114^  Martin, 2014^117^  Merkel, 2016^121^  Metzger, 1993^122^  Murray, 1996^131^  Mwenda, 2015^132^  Nazuk, 2018^134^  Nemeroff, 20001^35^  Oppe, 19901^41^  Paxton, 20071^48^  Perlmutter, 2013^149^  Perlmutter, 2015^150^  S. G. Shah, 2009^157^  Schow, 2006^158^  Strong, 2011^165^  Van Nieuwkoop, 2013^175^  Von Niederhäusern, 2017^181^  Yarborough, 2007^186^ | Aaron, 2017^1^  Abrams, 1986^2^  Achkar, 2003^4^  Anonymous, 2005^11^  Anonymous, 2005a^12^  Anonymous, 2006^14^  Awofeso, 2004^25^  Babor, 2009^26^  Barnes, 2017^30^  Barquera, 2017^33^  Baumrucker, 2012^36^  Brezis, 2008^39^  Carver, 2003^44^  Coates, 2009^49^  Couzin, 2005^55^  Day, 2007^59^  Eccles, 2012^64^  Glodé, 2002^77^  Greenwood, 2011^79^  Hester, 2010^90^  High, 1995^91^  Katz, 2014^101^  L. Wagner, 1990^106^  Lincoln., 2011^109^  Lynn, 1988^112^  M. S. Givel, 2000^115^  Macdonald, 1997^116^  Milne, 2012^123^  Morreim, 2011^126^  Muggli, 2003^129^  N. K. Shah, 2013^133^  Newton, 2016^136^  Nixon, 2015^138^  Obrist, 2015^139^  Orford, 2009^142^  Orlowski, 1996^143^  P. J. Adams, 2007^145^  Roehr, 2006^155^  Visseaux, 2013^179^  Wesson, 2013^183^ | Accili, 2015^3^  Ahmed, 2017^5^  Anonymous, 1999^8^  Anonymous, 2010^16^  Anonymous, 2011^17^  Anonymous, 2017 ^18^  Antonuccio, 2003^20^  Arie, 2014^22^  Baggott, 2008^27^  Bartek, 2014^34^  Batt, 2014^35^  Boivin, 2010^37^  Burton, 2005^40^  Cluzeau, 2012^48^  Cocchetto, 2008^50^  Colombo, 2014^51^  Colombo, 2012^53^  Daube, 2014^58^  Day, 2006^60^  Edgar, 2013^65^  FitzSimons, 2008^70^  Fowler, 2007^72^  Gabe., 2012^73^  Gasparini, 2006^74^  Gosden, 2001^78^  Grey, 2015^80^  Grouse, 2014^81^  Grouse, 2015^82^  Harbour, 2014^84^  Hayes, 2018^86^  Helms, 2015^88^  Herxheimer, 2003^89^  Hirst, 2003^92^  Hollak, 2016^94^  Hughes, 2013^95^  Johnson, 2004^98^  Johnston, 1998^99^  Kent, 2007^102^  Klemperer, 2009^103^  Korsia, 2000^104^  Kyriakides, 2009^105^  Lapsley, 2003^107^  Lin, 2017^108^  Lofgren, 2004^110^  McCarthy, 2017^118^  McCoy, 2016^119^  McCoy, 2018^120^  Mintzes, 2007^124^  Mosconi, 2003^127^  Moynihan, 2017^128^  Murphy, 2016^130^  O'Dowd, 2013^140^  P. Miller, 2009^146^  Patel, 2014^147^  Poot, 2009^151^  Raymond, 2010^154^  Rose, 2013^156^  Sheldon, 2010^159^  Slevin, 2006^160^  Smit, 2014^161^  Stein, 2018^162^  Steinbrook, 2016^163^  Superti-Furga, 2010^166^  Sweda, 1996^167^  Taylor, 2017^169^  Traulsen, 2005^171^  Tuffs, 2006^172^  Vandereycken, 2006^176^  Vermeulen, 2007^177^  Vernick, 1999^178^  Voelker, 2011^180^  Weinberg, 2011^182^  Williams, 2006^184^ | Apollonio, 2007^21^  Colombo, 2011^52^  Fallin, 2014^68^  Fooks, 2013^71^  M. Givel, 2007^113^  Nixon, 2015^137^  P. G. Miller, 2011^144^  R. B. Campbell, 2009^152^  R. Campbell, 2008^153^  Stone, 2004^164^  Tanenbaum, 2012^168^  Toiviainen, 2010^170^ | Baggott, 2014^28^  Lurie, 2006^111^  Morciano, 2016^125^  Van de Bovenkamp, 2015 ^174^ | **Full text not found**  Anonymous, 1974^7^  Anonymous, 1999a^9^  **Results not stratified by population group**  Atkins, 2013^24^  Hauser, 2017^85^  **Retracted article**  Barnoya, 2015^31^  **Full text not found**  Edwards, 1996^66^ |

**References**

1. Aaron DG, Siegel MB. Sponsorship of National Health Organizations by Two Major Soda Companies. *Am J Prev Med* 2017;52(1):20-30. doi: <https://dx.doi.org/10.1016/j.amepre.2016.08.010>

2. Abrams FR. Patient advocate or secret agent? *Jama* 1986;256(13):1784-5.

3. Accili D. Ceci n'est pas Science. *Cell Metabolism* 2015;21(4):503-04. doi: 10.1016/j.cmet.2015.03.011

4. Achkar E. Advocacy and public interest: are they compatible? *Am J Gastroenterol* 2003;98(3):527-9.

5. Ahmed AA, Yoo SK, Holliday EB, et al. Conflicts of interest among public speakers at the American society for radiation oncology annual meeting: Discrepancies reveal room for improvement. *International Journal of Radiation Oncology Biology Physics* 2017;99(2):E389. doi: 10.1016/j.ijrobp.2017.06.1534

6. Anderson P. The beverage alcohol industry's social aspects organizations: A public health warning. *Adicciones* 2003;15(2):103-14.

7. Anonymous. Water management and environment. IV. *TECHNEAU* 1974;No. 329:59-63.

8. Anonymous. Consumer group touts generics: Campaign uses Merck-Medco survey, funds. *American Journal of Health-System Pharmacy* 1999;56(22):2276-83.

9. Anonymous. Advocacy initiatives for African-Americans receive funding. *Body Posit* 1999;12(10):48-9.

10. Anonymous. Report raises questions about drug companies advertising budgets. *AIDS Policy Law* 1999;14(14):1, 6.

11. Anonymous. Public research and conflict of interest. *Medecine/Sciences* 2005;21(3):250.

12. Anonymous. Funding health advocacy. *Issue brief (Grantmakers Health)* 2005(21):1-35.

13. Anonymous. How to avoid future Vioxx-type scandals. *Prescrire International* 2005;14(77):115-7.

14. Anonymous. A look back at the pharmaceuticals market 2005. Deregulation continues. *Prescrire International* 2006;15(82):75-79.

15. Anonymous. Advocacy groups provide testing guidance for CDC funds. *AIDS Policy Law* 2007;22(20):5.

16. Anonymous. More clarity needed in funding of patient groups. *Prescrire International* 2010;19(105):43.

17. Anonymous. Drug companies and patient groups: the influence of funding. *Prescrire International* 2011;20(122):306.

18. Anonymous. Conflicts of interest in patient organizations: State of affairs in the US. *Geneesmiddelenbulletin* 2017;51(2):22-23.

19. Antarsh L. A patchwork of funding sources for Norplant users in the U.S. *AVSC News* 1992;30(3):1-2.

20. Antonuccio DO, Danton WG, McClanahan TM. Psychology in the prescription era: building a firewall between marketing and science. *Am Psychol* 2003;58(12):1028-43.

21. Apollonio DE, Bero LA. The creation of industry front groups: the tobacco industry and "get government off our back". *Am J Public Health* 2007;97(3):419-27.

22. Arie S, Mahony C. Should patient groups be more transparent about their funding? *Bmj* 2014;349:g5892. doi: <https://dx.doi.org/10.1136/bmj.g5892>

23. Armes DG. Mission informed discursive tactics of British mental health service-usersurvivor movement BSUSM resistance to formalization pressures accompanying contractual relationships with purchasing authorities. *Journal of Mental Health* 2009;18(4):344-52. doi: 10.1080/09638230802522973

24. Atkins L, Smith JA, Kelly MP, et al. The process of developing evidence-based guidance in medicine and public health: a qualitative study of views from the inside. *Implementation science* 2013;8(1):101.

25. Awofeso N. Five Ps in mix of public health advocacy. *Bmj* 2004;328(7431):110.

26. Babor TF. Alcohol research and the alcoholic beverage industry: issues, concerns and conflicts of interest. *Addiction* 2009;104 Suppl 1:34-47. doi: <https://dx.doi.org/10.1111/j.1360-0443.2008.02433.x>

27. Baggott R, Forster R. Health consumer and patients' organizations in Europe: towards a comparative analysis. *Health Expect* 2008;11(1):85-94. doi: <https://dx.doi.org/10.1111/j.1369-7625.2007.00472.x>

28. Baggott R, Jones K. The voluntary sector and health policy: The role of national level health consumer and patients' organisations in the UK. *Social Science and Medicine* 2014;123:202-09. doi: 10.1016/j.socscimed.2014.07.016

29. Baggott R, Jones KL. Representing Whom? U.K. Health Consumer and Patients' Organizations in the Policy Process. *J Bioeth Inq* 2018;25:25. doi: <https://dx.doi.org/10.1007/s11673-018-9859-4>

30. Barnes B. Financial conflicts of interest in continuing medical education: Implications and accountability. *JAMA - Journal of the American Medical Association* 2017;317(17):1741-42. doi: 10.1001/jama.2017.2981

31. Barnoya J, Nestle M. The food industry and conflicts of interest in nutrition research: A Latin American perspective.[Retraction in J Public Health Policy. 2016 Feb;37(1):126-7; PMID: 26658288]. *J Public Health Policy* 2015;29:29. doi: <https://dx.doi.org/10.1057/jphp.2015.37>

32. Barohn RJ. Why patient-centered outcomes research? *Journal of Neuromuscular Diseases* 2016;3:S50-S51. doi: 10.3233/JND-160001

33. Barquera S. What the world can learn from Mexico's battle against obesity: Vulnerable groups, international collaboration, evidence-based policies and management of conflicts of interest. *Annals of Nutrition and Metabolism* 2017;71:14. doi: 10.1159/000480486

34. Bartek RJ. Foundation-industry relationships--a new business model joint-venture philanthropy in therapy development. *Curr Top Med Chem* 2014;14(3):313-8.

35. Batt S. Who will support independent patient groups? *BMJ (Online)* 2014;349 doi: 10.1136/bmj.g6306

36. Baumrucker SJ, Adkins RW, Stolick M, et al. Surrogates with conflicting interests: who makes the decision? *Am J Hosp Palliat Care* 2012;29(6):497-500. doi: <https://dx.doi.org/10.1177/1049909112451104>

37. Boivin A, Currie K, Fervers B, et al. Patient and public involvement in clinical guidelines: international experiences and future perspectives. *BMJ Quality & Safety* 2010:qshc. 2009.034835.

38. Bramstedt KA. Questioning the decision-making capacity of surrogates. *Intern Med J* 2003;33(5-6):257-9.

39. Brezis M. Big pharma and health care: unsolvable conflict of interests between private enterprise and public health. *Isr J Psychiatry Relat Sci* 2008;45(2):83-9; discussion 90-4.

40. Burton B. Drug companies told that sponsoring patients' groups might help win approval for their products. *Bmj* 2005;331(7529):1359.

41. Cahill J. Are you prepared to be their advocate? Issues in patient advocacy. *Prof Nurse* 1994;9(6):371-2, 74-5.

42. Carleton BC. From professional advocacy to patient advocacy: a gambit for pharmacists. *Journal of the American Pharmaceutical Association (Washington,DC : 1996)* 2001;41(1):17-18.

43. Carroll-Johnson RM. Entitlement. *Oncol Nurs Forum* 2003;30(3):355.

44. Carver V, Reinert B, Range LM, et al. Nonprofit organizations versus government agencies to reduce tobacco use. *J Public Health Policy* 2003;24(2):181-94.

45. Charters MA. The patient representative role and sources of power. *Hosp Health Serv Adm* 1993;38(3):429-42.

46. Cheson BD. Letter from the editor. *Clinical Advances in Hematology and Oncology* 2010;8(5):1-1.

47. Clements CJ, Ratzan S. Misled and confused? Telling the public about MMR vaccine safety. Measles, mumps, and rubella. *J Med Ethics* 2003;29(1):22-6.

48. Cluzeau F, Wedzicha JA, Kelson A, et al. Stakeholder involvement: How to do it right: Article 9 in integrating and coordinating efforts in COPD guideline development. An official ATS/ERS workshop report. *Proceedings of the American Thoracic Society* 2012;9(5):269-73. doi: 10.1513/pats.201208-062ST

49. Coates AS, Goldhirsch A, Gelber RD, et al. Reply to Does the expert panel at the St Gallen meeting provide an unbiased opinion about the management of women with early breast cancer? *Annals of Oncology* 2009;20(10):1751-52. doi: 10.1093/annonc/mdp400

50. Cocchetto DM, Hassall TH, Carter L, et al. Best practices for the Advisory Committee process for products regulated by CDER and CBER: A PhRMA white paper. *Drug Information Journal* 2008;42(1):13-21.

51. Colombo C, Mosconi P. Transparency of funding of patient groups is mandatory but is not enough. *Bmj* 2014;349:g6301. doi: <https://dx.doi.org/10.1136/bmj.g6301>

52. Colombo C, Clavenna A, Bonati M, et al. Does research respond to patients' needs? A survey of disable patient-led paediatric associations. *Ricerca e Pratica* 2011;27(4):135-43.

53. Colombo C, Moja L, Gonzalez-Lorenzo M, et al. Patient empowerment as a component of health system reforms: Rights, benefits and vested interests. *Internal and Emergency Medicine* 2012;7(2):183-87. doi: 10.1007/s11739-012-0757-1

54. Conter HJ. Patient advocacy: Health, money or both? *CMAJ* 2009;181(11):825. doi: 10.1503/cmaj.109-2032

55. Couzin J. Clinical research. Advocating, the clinical way. *Science* 2005;308(5724):940-2.

56. Cukor D, Cohen LM, Cope EL, et al. Patient and other stakeholder engagement in patient-centered outcomes research institute funded studies of patients with kidney diseases. *Clinical Journal of the American Society of Nephrology* 2016;11(9):1703-12. doi: 10.2215/CJN.09780915

57. Dang A, Likhar N, Vsn M, et al. Patient advocacy and changing paradigm in drug access. *Value in Health* 2016;19(3):A294.

58. Daube M. Protecting their paymasters. *Addiction (Abingdon, England)* 2014;109(4):526-27. doi: 10.1111/add.12416

59. Day M, Boseley S. The World Health Organization, the drugs company, and the $10,000 funding offer. *Int J Health Serv* 2007;37(3):589-90.

60. Day M. UK drug companies must disclose funding of patients' groups. *Bmj* 2006;332(7533):69.

61. Dellasega C. A piece of my mind. Conflict of interest. *Jama* 2003;290(19):2521-2.

62. Di Pietro NC, Whiteley L, Illes J. Treatments and services for neurodevelopmental disorders on advocacy websites: Information or evaluation? *Neuroethics* 2012;5(2):197-209. doi: 10.1007/s12152-011-9102-z

63. Miller EA, Pole A, Usidame B. Life and death in the mental-health blogosphere: An analysis of blog content and survival. *World Medical and Health Policy* 2015;7(1):59-82. doi: 10.1002/wmh3.133

64. Eccles MP, Grimshaw JM, Shekelle P, et al. Developing clinical practice guidelines: target audiences, identifying topics for guidelines, guideline group composition and functioning and conflicts of interest. *Implement Sci* 2012;7:60. doi: <https://dx.doi.org/10.1186/1748-5908-7-60>

65. Edgar A. The dominance of big pharma: power. *Med Health Care Philos* 2013;16(2):295-304. doi: <https://dx.doi.org/10.1007/s11019-012-9385-9>

66. Edwards SS, Vinicky JK, Orlowski JP. Conflicts of interest, conflicting interests, and interesting conflicts, part 2. *J Clin Ethics* 1996;7(1):69-76.

67. Entwistle VA, O'Donnell M. Research funding organisations and consumer involvement. *J Health Serv Res Policy* 2003;8(3):129-31.

68. Fallin A, Grana R, Glantz SA. 'To quarterback behind the scenes, third-party efforts': the tobacco industry and the Tea Party. *Tob Control* 2014;23(4):322-31. doi: <https://dx.doi.org/10.1136/tobaccocontrol-2012-050815>

69. Fentiman LC. Patient advocacy and termination from managed care organizations. Do state laws protecting health care professional advocacy make any difference? *Neb Law Rev* 2003;82(2):508-74.

70. FitzSimons DW. Prevention and control of viral hepatitis: the role and impact of patient and advocacy groups in and outside Europe. *Vaccine* 2008;26(45):5669-74. doi: <https://dx.doi.org/10.1016/j.vaccine.2008.08.023>

71. Fooks GJ, Gilmore AB. Corporate philanthropy, political influence, and health policy. *PLoS ONE* 2013;8(11):e80864. doi: <https://dx.doi.org/10.1371/journal.pone.0080864>

72. Fowler C. Contentious issue of funding and the pharmaceutical industry--should service user groups take the big pharma shilling, and and what are the costs if they do? *Ment Health Today* 2007:40.

73. Gabe J, Chamberlain K, Norris P, et al. The debate about the funding of Herceptin: a case study of 'countervailing powers'. *Soc Sci Med* 2012;75(12):2353-61. doi: <https://dx.doi.org/10.1016/j.socscimed.2012.09.009>

74. Gasparini M, Bonito V, Leonardi M, et al. Neurologists and patient's associations: Alliances and conflicts. *Neurological Sciences* 2006;27(3):194-204. doi: 10.1007/s10072-006-0669-9

75. Gillam S, Newbould J. Patient participation groups in general practice: What are they for, where are they going? *BMJ (Online)* 2016;352 doi: 10.1136/bmj.i673

76. Gillen D. The pharmaceutical industry, the NHS and academic medicine: Time for a new relationship? *Basic and Clinical Pharmacology and Toxicology* 2009;105:7. doi: 10.1111/j.1742-7843.2009.00435.x

77. Glodé ER. Advising under the influence?: Conflicts of interest among FDA advisory committee members. *Food and Drug Law Journal* 2002;57(2):293-322+iii.

78. Gosden R, Beder S. Pharmaceutical industry agenda setting in mental health policies. *Ethical hum* 2001;3(3):147-59.

79. Greenwood S. What price independence? *Australian Journal of Pharmacy* 2011;92(1098):16-17.

80. Grey A, Bolland M. Web of industry, advocacy, and academia in the management of osteoporosis. *Bmj* 2015;351:h3170. doi: <https://dx.doi.org/10.1136/bmj.h3170>

81. Grouse L. ICC policy statement concerning ICC sponsorship funds. *Journal of Thoracic Disease* 2014;6(9):E202. doi: 10.3978/j.issn.2072-1439.2014.09.02

82. Grouse L. Gary Bain's precepts for chronic obstructive pulmonary disease patient groups. *Journal of Thoracic Disease* 2015;7(4):E79-E80. doi: 10.3978/j.issn.2072-1439.2015.04.24

83. Hale MM. Dealing with the conflict of interests in health care reform. *J Health Hum Serv Adm* 1998;21(2):162-80.

84. Harbour RT. What about non-financial conflicts of interest? *BMJ (Online)* 2014;348 doi: 10.1136/bmj.g1154

85. Hauser W, Petzke F, Kopp I, et al. [Impact of conflicts of interest on guideline recommendations : Empirical study within the second update of the German interdisciplinary S3 guidelines on fibromyalgia syndrome]. *Schmerz* 2017;31(3):308-18. doi: <https://dx.doi.org/10.1007/s00482-017-0218-x>

86. Hayes MJ, Prasad V. Financial Conflicts of Interest at FDA Drug Advisory Committee Meetings. *Hastings Cent Rep* 2018;48(2):10-13. doi: <https://dx.doi.org/10.1002/hast.833>

87. Hays R, Matas AJ. Ethical review of the responsibilities of the patient advocate in living donor liver transplant. *Clinical Liver Disease* 2016;7(3):57-59. doi: 10.1002/cld.533

88. Helms U, Klemperer D. Patient self-help. Conflicts of interest by pharmaceutical sponsorship. *Internistische Praxis* 2015;55(1):173-79.

89. Herxheimer A. Relationships between the pharmaceutical industry and patients' organisations. *Bmj* 2003;326(7400):1208-10.

90. Hester JB. Competing interests and the British Pain Society. *BMJ (Online)* 2010;340(7740):227. doi: 10.1136/bmj.c476

91. High DM, Doole MM. Ethical and legal issues in conducting research involving elderly subjects. *Behav Sci Law* 1995;13(3):319-35.

92. Hirst J. Charities and patient groups should declare interests. *Bmj* 2003;326(7400):1211.

93. Hofmann B, Saarni SI. Who can and who should represent the patient? *International Journal of Technology Assessment in Health Care* 2011;27(4):403. doi: 10.1017/S0266462311000353

94. Hollak CE, Biegstraaten M, Baumgartner MR, et al. Position statement on the role of healthcare professionals, patient organizations and industry in European Reference Networks. *Orphanet J Rare Dis* 2016;11:7. doi: <https://dx.doi.org/10.1186/s13023-016-0383-5>

95. Hughes D, Williams-Jones B. Coalition Priorite Cancer and the pharmaceutical industry in Quebec: conflicts of interest in the reimbursement of expensive cancer drugs? *Healthc Policy* 2013;9(1):52-64.

96. Wagner JA. Open mind, open collaboration. *Biomarkers in Medicine* 2011;5(6):701-03. doi: 10.2217/bmm.11.93

97. Jaklevic MC. Identity crisis. Advocacy group called front for insurance industry. *Mod Healthc* 2004;34(13):6-7.

98. Johnson LJ. The risks of being a "patient advocate". *Med Econ* 2004;81(8):72.

99. Johnston K. Patient advocates or patient adversaries? Using fiduciary law to compel disclosure of managed care financial incentives. *San Diego Law Rev* 1998;35(4):951-92.

100. Kapp MB. Who's the client? Complex conundrum in a context of conflicting interests. *J Ethics Law Aging* 1999;5(2):95-101.

101. Katz LJ. How will transparency redefine the relationship between physicians, patients, and the medical industry? *Survey of Ophthalmology* 2014;59(3):345. doi: 10.1016/j.survophthal.2013.09.001

102. Kent A. Should patient groups accept money from drug companies? Yes. *Bmj* 2007;334(7600):934.

103. Klemperer D. [Self-help groups conflicts of interest through sponsoring by the pharmaceutical industry]. *Bundesgesundheitsblatt Gesundheitsforschung Gesundheitsschutz* 2009;52(1):71-6. doi: <https://dx.doi.org/10.1007/s00103-009-0750-7>

104. Korsia S. Partnerships between the pharmaceutical industry and patient groups: The patients' view. *Drug Information Journal* 2000;34(1):79-81.

105. Kyriakides S. Patient groups - Meeting the challenge of sustainable funding. *European Journal of Cancer, Supplement* 2009;7(2-3):6.

106. Wagner L. Drug marketing practices criticized. *Mod Healthc* 1990;20(50):12.

107. Lapsley PM. Industry funding of patients' support groups: objectives of patients, clinicians, and industry are similar. *Bmj* 2003;327(7410):343-4.

108. Lin DH, Lucas E, Murimi IB, et al. Financial conflicts of interest and the centers for disease control and prevention's 2016 guideline for prescribing opioids for chronic pain. *Pharmacoepidemiology and Drug Safety* 2017;26:213-14. doi: 10.1002/pds.4275

109. Lincoln P, Rundall P, Jeffery B, et al. Conflicts of interest and the un high-level meeting on non-communicable diseases. *The Lancet* 2011;378(9804):e6. doi: 10.1016/S0140-6736(11)61463-3

110. Lofgren H. Pharmaceuticals and the consumer movement: the ambivalences of 'patient power'. *Aust Health Rev* 2004;28(2):228-37.

111. Lurie P, Almeida CM, Stine N, et al. Financial conflict of interest disclosure and voting patterns at Food and Drug Administration Drug Advisory Committee meetings. *Jama* 2006;295(16):1921-28.

112. Lynn J. Conflicts of interest in medical decision-making. *J Am Geriatr Soc* 1988;36(10):945-50.

113. Givel M. Consent and counter-mobilization: the case of the national smokers alliance. *J Health Commun* 2007;12(4):339-57.

114. Adams MP. Dual agency and role morality. *Am J Bioeth* 2014;14(9):44-5. doi: <https://dx.doi.org/10.1080/15265161.2014.936249>

115. Givel MS, Glantz SA. Failure to defend a successful state tobacco control program: policy lessons from Florida. *Am J Public Health* 2000;90(5):762-7.

116. Macdonald H, Aguinaga S, Glantz SA. The defeat of Philip Morris' 'California Uniform Tobacco Control Act'. *Am J Public Health* 1997;87(12):1989-96.

117. Martin J, Cook R. Harnessing coalitions to counter food industry solutions-the Coca Cola journey. *Asia-Pacific Journal of Clinical Oncology* 2014;10:215. doi: 10.1111/ajco.12332

118. McCarthy M. More than 80% of US patients' groups take industry funds, study finds. *Bmj* 2017;356:j1180. doi: <https://dx.doi.org/10.1136/bmj.j1180>

119. McCoy MS, Emanuel EJ. Health policy: Addressing conflicts of interest of public speakers at advisory committee meetings. *Nat Rev Clin Oncol* 2016;13(5):267-8. doi: <https://dx.doi.org/10.1038/nrclinonc.2016.54>

120. McCoy MS. Industry Support of Patient Advocacy Organizations: The Case for an Extension of the Sunshine Act Provisions of the Affordable Care Act. *Am J Public Health* 2018;108(8):1026-30. doi: <https://dx.doi.org/10.2105/AJPH.2018.304467>

121. Merkel PA, Manion M, Gopal-Srivastava R, et al. The partnership of patient advocacy groups and clinical investigators in the rare diseases clinical research network. *Orphanet J Rare Dis* 2016;11(1) doi: 10.1186/s13023-016-0445-8

122. Metzger D, Strand VC. Violence prevention: Trends in foundation funding. *Health Affairs* 1993;12(4):209-20.

123. Milne C. Defining conflict of interest. *Pharmaceutical Technology* 2012;36(3):38.

124. Mintzes B. Should patient groups accept money from drug companies? No. *Bmj* 2007;334(7600):935.

125. Morciano C, Basevi V, Faralli C, et al. Policies on conflicts of interest in health care guideline development: a cross-sectional analysis. *PloS one* 2016;11(11):e0166485.

126. Morreim EH. Taking a lesson from the lawyers: defining and addressing conflict of interest. *Am J Bioeth* 2011;11(1):33-4. doi: <https://dx.doi.org/10.1080/15265161.2010.540064>

127. Mosconi P. Industry funding of patients' support groups: declaration of competing interests is rare in Italian breast cancer associations. *Bmj* 2003;327(7410):344.

128. Moynihan R, Bero L. Toward a Healthier Patient Voice: More Independence, Less Industry Funding. *JAMA Internal Medicine* 2017;177(3):350-51. doi: <https://dx.doi.org/10.1001/jamainternmed.2016.9179>

129. Muggli ME, Hurt RD. Tobacco industry strategies to undermine the 8th World Conference on Tobacco or Health. *Tob Control* 2003;12(2):195-202.

130. Murphy E, Fukui A, Beuamont A. Mapping transactions from pharmaceutical companies to healthcare organisations in the EU5. *Value in Health* 2016;19(7):A439.

131. Murray S. Care in the community: a conflict of interests? *Bmj* 1996;313(7057):632.

132. Mwenda SA, Mwachiro MD. The surgeon and advocacy. *Annals of African Surgery* 2015;12(1):1-3.

133. Shah NK. Corporate philanthropy and conflicts of interest in public health: ExxonMobil, Equatorial Guinea, and malaria. *J Public Health Policy* 2013;34(1):121-36. doi: <https://dx.doi.org/10.1057/jphp.2012.60>

134. Nazuk A, Shabbir J. A new disclosure index for Non-Governmental Organizations. *PLoS ONE* 2018;13(2):e0191337. doi: <https://dx.doi.org/10.1371/journal.pone.0191337>

135. Nemeroff CB. The role of patient advocacy in funding policy. *CNS Spectr* 2000;5(3):17.

136. Newton A, Lloyd-Williams F, Bromley H, et al. Food for thought? Potential conflicts of interest in academic experts advising government and charities on dietary policies. *BMC Public Health* 2016;16:735. doi: <https://dx.doi.org/10.1186/s12889-016-3393-2>

137. Nixon L, Mejia P, Cheyne A, et al. Big Soda’s long shadow: news coverage of local proposals to tax sugar-sweetened beverages in Richmond, El Monte and Telluride. *Critical Public Health* 2015;25(3):333-47. doi: 10.1080/09581596.2014.987729

138. Nixon L, Mejia P, Cheyne A, et al. "We're Part of the Solution": Evolution of the Food and Beverage Industry's Framing of Obesity Concerns Between 2000 and 2012. *Am J Public Health* 2015;105(11):2228-36. doi: 10.2105/AJPH.2015.302819

139. Obrist R. Only conflicts of interest? *Swiss Medical Weekly* 2015;145 doi: 10.4414/smw.2015.14120

140. O'Dowd A. Drug trade bodies rally patient groups to deflect calls for full trial data. *Bmj* 2013;347:f4702. doi: <https://dx.doi.org/10.1136/bmj.f4702>

141. Oppe TE. Ethical aspects of AIDS in childhood in England. *Pediatrician* 1990;17(2):115-7.

142. Orford J. Governments as promoters of dangerous consumptions. *Addiction* 2009;104(5):693-95. doi: 10.1111/j.1360-0443.2008.02495.x

143. Orlowski JP, Vinicky JK, Edwards SS. Conflicts of interest, conflicting interests, and interesting conflicts, Part 3. *J Clin Ethics* 1996;7(2):184-6.

144. Miller PG, de Groot F, McKenzie S, et al. Vested interests in addiction research and policy. Alcohol industry use of social aspect public relations organizations against preventative health measures. *Addiction* 2011;106(9):1560-7. doi: <https://dx.doi.org/10.1111/j.1360-0443.2011.03499.x>

145. Adams PJ. Assessing whether to receive funding support from tobacco, alcohol, gambling and other dangerous consumption industries. *Addiction* 2007;102(7):1027-33.

146. Miller P, kypri K. Why we will not accept funding from Drinkwise. *Drug and Alcohol Review* 2009;28(3):324-26. doi: 10.1111/j.1465-3362.2009.00072.x

147. Patel NC. Successful implementation of a local advocacy group for primary immunodeficiency diseases. *Journal of Clinical Immunology* 2014;34(3):383. doi: 10.1007/s10875-014-0013-8

148. Paxton S, Stephens D. Challenges to the meaningful involvement of HIV-positive people in the response to HIV/AIDS in Cambodia, India and Indonesia. *Asia-Pacific Journal of Public Health* 2007;19(1):8-13. doi: 10.1177/10105395070190010301

149. Perlmutter J, Bell SK, Darien G. Cancer research advocacy: Past, present, and future. *Cancer Research* 2013;73(15):4611-15. doi: 10.1158/0008-5472.CAN-12-4553-T

150. Perlmutter J, Roach N, Smith ML. Involving Advocates in Cancer Research. *Semin Oncol* 2015;42(5):681-5. doi: <https://dx.doi.org/10.1053/j.seminoncol.2015.07.008>

151. Poot E. Why does the industry need a code of practise on cooperation with patient organisations? *Molecular Human Reproduction* 2009;24:i59. doi: 10.1093/humrep/dep738

152. Campbell RB, Balbach ED. Building alliances in unlikely places: progressive allies and the Tobacco Institute's coalition strategy on cigarette excise taxes. *Am J Public Health* 2009;99(7):1188-96. doi: <https://dx.doi.org/10.2105/AJPH.2008.143131>

153. Campbell R, Balbach ED. Mobilising public opinion for the tobacco industry: the Consumer Tax Alliance and excise taxes. *Tob Control* 2008;17(5):351-6. doi: <https://dx.doi.org/10.1136/tc.2008.025338>

154. Raymond G. Who funds patient groups? *Prescrire International* 2010;19(111):293.

155. Roehr B. US national academies' panellists are accused of financial conflicts. *BMJ (Clinical research ed)* 2006;333(7561):219.

156. Rose SL. Patient advocacy organizations: institutional conflicts of interest, trust, and trustworthiness. *J Law Med Ethics* 2013;41(3):680-7. doi: <https://dx.doi.org/10.1111/jlme.12078>

157. Shah SG, Farrow A, Robinson I. The representation of healthcare end users' perspectives by surrogates in healthcare decisions: a literature review. *Scand J Caring Sci* 2009;23(4):809-19. doi: <https://dx.doi.org/10.1111/j.1471-6712.2008.00674.x>

158. Schow D. The culture of domestic violence advocacy: values of equality/behaviors of control. *Women Health* 2006;43(4):49-68.

159. Sheldon T. Patient groups must reveal corporate sponsorship, urges campaign group. *Bmj* 2010;341:c4459. doi: <https://dx.doi.org/10.1136/bmj.c4459>

160. Slevin M. Funding of patients' groups. *Lancet* 2006;368(9531):202.

161. Smit C. Personal Reflections of a Patient Representative in an Appraisal Committee. *Patient* 2014;8(1):5-10. doi: 10.1007/s40271-014-0086-8

162. Stein S, Bogard E, Boice N, et al. Principles for interactions with biopharmaceutical companies: the development of guidelines for patient advocacy organizations in the field of rare diseases. *Orphanet J Rare Dis* 2018;13(1):18. doi: <https://dx.doi.org/10.1186/s13023-018-0761-2>

163. Steinbrook R. The Financial Associations of Public Speakers at Meetings of Federal Health Advisory Committees. *JAMA Internal Medicine* 2016;176(3):391. doi: <https://dx.doi.org/10.1001/jamainternmed.2015.8092>

164. Stone M, Siegel MB. Tobacco industry sponsorship of community-based public health initiatives: why AIDS and domestic violence organizations accept or refuse funds. *J Public Health Manag Pract* 2004;10(6):511-7.

165. Strong D, Lipson D, Honeycutt T, et al. Foundation's consumer advocacy health reform initiative strengthened groups' effectiveness. *Health Affairs* 2011;30(9):1799-803. doi: <https://dx.doi.org/10.1377/hlthaff.2011.0551>

166. Superti-Furga A, Garavelli L. Current themes in molecular pediatrics: molecular medicine and its applications. *Ital* 2010;36:20. doi: <https://dx.doi.org/10.1186/1824-7288-36-20>

167. Sweda EL, Jr., Daynard RA. Tobacco industry tactics. *Br Med Bull* 1996;52(1):183-92.

168. Tanenbaum SJ. Consumer-operated service organizations: organizational characteristics, community relationships, and the potential for citizenship. *Community Ment Health J* 2012;48(4):397-406. doi: <https://dx.doi.org/10.1007/s10597-011-9408-7>

169. Taylor J, Denegri S. Industry links with patient organisations. *Bmj* 2017;356:j1251. doi: <https://dx.doi.org/10.1136/bmj.j1251>

170. Toiviainen HK, Vuorenkoski LH, Hemminki EK. Patient organizations in Finland: increasing numbers and great variation. *Health Expect* 2010;13(3):221-33. doi: <https://dx.doi.org/10.1111/j.1369-7625.2008.00499.x>

171. Traulsen JM, Almarsdottir AB. Pharmaceutical policy and the lay public. *Pharm World Sci* 2005;27(4):273-7.

172. Tuffs A. Sponsorship of patients' groups by drug companies should be made transparent. *Bmj* 2006;333(7581):1238.

173. Van de Bovenkamp HM, Trappenburg MJ. Government influence on patient organizations. *Health Care Anal* 2011;19(4):329-51. doi: <https://dx.doi.org/10.1007/s10728-010-0155-7>

174. van de Bovenkamp HM, Zuiderent‐Jerak T. An empirical study of patient participation in guideline development: exploring the potential for articulating patient knowledge in evidence‐based epistemic settings. *Health Expectations* 2015;18(5):942-55.

175. Van Nieuwkoop L, Poot S, Sauer R, et al. How to develop an independent youth organisation? Risks, challenges and benefits. *Annals of the Rheumatic Diseases* 2013;72 doi: 10.1136/annrheumdis-2013-eular.3192

176. Vandereycken W. [Bitter and gilded pills: psychiatry in the light (or shadow) of the pharmaceutical industry]. *Tijdschr Psychiatr* 2006;48(2):119-29.

177. Vermeulen M, Bouma J. [The influence of the pharmaceutical industry in patient organisations]. *Ned Tijdschr Geneeskd* 2007;151(44):2432-4.

178. Vernick JS. Lobbying and advocacy for the public's health: what are the limits for nonprofit organizations? *Am J Public Health* 1999;89(9):1425-9.

179. Visseaux G, Clément R. Legal expertise and conflicts of interest. *Medecine et Droit* 2013;2013(119):36-43. doi: 10.1016/j.meddro.2013.02.001

180. Voelker R. Study: Few advocacy groups disclose grants from drug companies. *Jama* 2011;305(7):662. doi: <https://dx.doi.org/10.1001/jama.2011.119>

181. Von Niederhäusern B, Schandelmaier S, Bonde MM, et al. Towards the development of a comprehensive framework: Qualitative systematic survey of definitions of clinical research quality. *PLoS ONE* 2017;12(7) doi: 10.1371/journal.pone.0180635

182. Weinberg M. Patient advocacy organizations and corporate relationships. *Am J Public Health* 2011;101(4):582-3; author reply 83. doi: <https://dx.doi.org/10.2105/AJPH.2011.300087>

183. Wesson DE. An organizational approach to conflicts of interest: lessons from non-health care businesses. *JAMA Internal Medicine* 2013;173(16):1489-90. doi: <https://dx.doi.org/10.1001/jamainternmed.2013.8897>

184. Williams HC, Naldi L, Paul C, et al. Conflicts of interest in dermatology. *Acta Derm Venereol* 2006;86(6):485-97.

185. Williamson RT, Rodd J. Civil society advocacy in Nigeria: promoting democratic norms or donor demands? *BMC Int Health Hum Rights* 2016;16(1):19. doi: <https://dx.doi.org/10.1186/s12914-016-0093-z>

186. Yarborough M, Sharp RR. Bioethics consultation and patient advocacy organizations: expanding the dialogue about professional conflicts of interest. *Camb Q Healthc Ethics* 2007;16(1):74-81.
