# Supplementary material for: Financial relationships between patient and consumer representatives and the health industry: A systematic review
Source: Health Expect. 2019 Dec 19;23(2):483–95. doi: 10.1111/hex.13013 (PMC7104632; doi:10.1111/hex.13013)
Supplement: Supplementary file 4 [file HEX-23-483-s004.docx]

**Appendix 4: Characteristics of included studies addressing patients’ organizations (N=20)**

| *Author Year* | *Research methodology* | *Organizations, focus of the study and country* | *Data collected and source* | *Outcome related to organization* | *Outcome related to members* | *Funding of the study* | *COI of study authors* |
| --- | --- | --- | --- | --- | --- | --- | --- |
| O’Donovan 2007[^1^](#_ENREF_1) | Mixed methods | - **Organizations:** 112 Irish ‘health advocacy organizations’ meeting inclusion criteria from a compiled database - **Focus:** pharmaceutical industry - **Country:** Ireland | - **Reported funding:** postal and e-mail questionnaire (2004) - **Qualitative data:** interviews (2005) and analyses of documentation published by and about the organizations, conferences and seminars hosted by the organizations (ethnographic research) | - Receiving funding - Policy for dealing with industry - Categorization of the relationship between organizations and the industry - Justification for patient organizations’ cooperation with the industry | ---- | Royal Irish Academy’s Third Sector Research Programme | Not reported |
| Pinto 2016,[^2^](#_ENREF_2) Pinto 2017[^3^](#_ENREF_3) | Quantitative^[[1]](#footnote-1)^ | - **Organizations:** 61 Australian rare disease ‘patient organizations’ identified 1) from the websites of two Australian rare disease alliance groups^[[2]](#footnote-2)^, 2) from the Australian Charities and Not for Profits Register, 3) using a snowballing method; excluded organizations without a website - **Focus:** industry (pharma or biotech company) - **Country:** Australia | - **Reported funding:** online questionnaire (2013 – 2014) - **Acknowledged funding:** survey of websites | - Receiving funding - Acknowledging funding | ---- | Not funded | None |
| Li 2019[^4^](#_ENREF_4) | Quantitative | - **Organizations:** 24 dermatology ‘patient advocacy organizations’ with a reported annual revenue >$500,000 obtained from the Kaiser Health News database; excluded organizations with invalid tax Forms 990 or website - **Focus:** industry (drug, device and biotechnology) - **Country:** U.S. | - **Reported funding:** survey of websites and 2015 annual reports - **Total revenue of organizations:** survey of 2015 federal tax Forms 990 - **Employment of board members:** survey of websites of organizations and LinkedIn search | - Receiving funding - Reporting on funding amount - Funding amount - Proportion of total donations attributable to funding source - Reporting on direction of use of funding - Reporting on identity of funders | - Employment of board members by the industry | National Center for Advancing Translational Sciences, National Institutes of Health | “None reported” |
| Lexchin 2019[^5^](#_ENREF_5) | Quantitative | - **Organizations:** 372 submissions to CDR and pCODR^[[3]](#footnote-3)^ from 93 Canadian ‘patient groups’ - **Focus:** pharmaceutical industry - **Country:** Canada | - **Reported funding:** Survey of submissions about funding of particular drug indications to the CDR and pCODR3 (2018) - **Views of organizations:** analysis of submission extracts by the authors | - Receiving funding - Funding amount - Reporting on proportion of annual budget attributable to funding source - Proportion of annual budget attributable to funding source - Reporting on direction of use of funding - Direction of use of funding - Effect of funding (association between funding and views of organizations) - Median number of conflicts per submission | ---- | Not funded | 1/1 paid consultant^[[4]](#footnote-4)^, paid panel member^[[5]](#footnote-5)^, member of research groups receiving money from the CIHR^[[6]](#footnote-6)^ and the Australian NHMRC^[[7]](#footnote-7)^, member of the Foundation Board of HAI^[[8]](#footnote-8)^ and the Board of Canadian Doctors for Medicare |
| Fabbri 2019[^6^](#_ENREF_6) | Quantitative | - **Organizations:** 230 ‘health consumer organizations’ in Australia (34 pharmaceutical companies providing 1,487 sponsorships) - **Focus:** pharmaceutical industry - **Country:** Australia | - **Funding:** Survey of 2013 – 2016 Medicines Australia reports - **Determining clinical areas of focus:** Survey of Australian Charities and Nonprofits Commission website and Internet search - **Identifying whether pharmaceutical companies had drugs on the market for conditions covered by the consumer organization:** Survey of websites pharmaceutical companies providing funding for most funded consumer organizations - **Identifying medicines under review:** Survey of 2013 – 2016 PBS^[[9]](#footnote-9)^, PBAC^[[10]](#footnote-10)^ public summary documents | - Funding amount - Number of sponsorships - Direction of use of funding (including unrestricted funding) - Alignment between funding and industry’s interests | ---- | Partially funded via a University of Sydney–Charles Perkins Centre summer scholarship | 1/5 - expert witness on behalf of plaintiffs in a Canadian class action suit concerning cardiovascular risks of a testosterone gel |
| Mandeville 2019[^7^](#_ENREF_7) | Quantitative | - **Organizations:** 53 ‘patient organizations’ contributing to 41 NICE^[[11]](#footnote-11)^ technology appraisals on 117 separate occasions - **Focus:** pharmaceutical, device, and/or biotechnology companies - **Country:** UK | - **Reported funding (as reported by the organizations):** Survey of websites, accounts and annual reports of patient organizations, website of Charity Commission for England and Wales and the Scottish Charity Regulator, contacting patient organizations by email or website enquiry form - **Reported funding (as reported by the pharmaceutical industry):** Survey of ABPI’s^[[12]](#footnote-12)^ Disclosure UK database, survey of websites of pharmaceutical companies - **Disclosed funding:** Survey of published documents pertaining to 2015-2016 technology appraisals, survey of nomination forms held by NICE^1^1 | - Receiving funding (disclosed and undisclosed) - Funding amount - Proportion of income attributable to funding source - Number of funders - Policy for dealing with industry | ---- | Not funded; “one author is an employee of NICE1^1^” | 1/6 employee of NICE1^1^, chair of trustees for a local branch of a national patient organization (Mind; has a policy that restricts staff and trustees from receiving funding from pharmaceutical companies); 1/6 undertook a specialty training placement at NICE1^1^ before starting this work |
| Peterlein 2018[^8^](#_ENREF_8) | Quantitative | - **Organizations:** 36 ‘self-help groups’ in the field of pediatric orthopedics in Germany - **Focus:** pharmaceutical industry - **Country:** Germany | - **Reported funding:** postal questionnaire (2015) | - Receiving funding | ---- | Not reported | None |
| Abola 2016[^9^](#_ENREF_9) | Quantitative | - **Organizations:** 68 ‘patient advocacy organizations’ listed on the NCCN^[[13]](#footnote-13)^’s patient advocacy webpage (NCCN^13^ website accessed: Dec 2015) - **Focus:** biopharmaceutical industry - **Country:** U.S. | - **Reported funding:** survey of organizations’ websites (2015 – 2016) | - Receiving funding - Number of funders - Policy for dealing with industry | ---- | Not reported | Not reported |
| Ball 2006[^10^](#_ENREF_10) | Quantitative | - **Organizations:** 69 national and international ‘patient organizations’ based in the USA, UK, Australia, Canada, and South Africa for 10 major health conditions^[[14]](#footnote-14)^ - **Focus:** pharmaceutical industry - **Country:** international (USA, UK, Australia, Canada, and South Africa) | - **Reported funding and total revenue:** survey of websites and annual reports (website identification: 2005; website assessment: 2006); contacted organizations that did not report on pharmaceutical funding | - Receiving funding - Reporting on funding amount - Reporting on proportion of annual income attributable to funding source - Reporting on direction of use of funding - Direction of use of funding (including unrestricted funding) - Number of funders | ---- | Not funded | 3/3 members of a health advocacy organization |
| Rose 2017[^11^](#_ENREF_11) | Quantitative | - **Organizations:** 289 leaders from a nationally representative random sample of ‘patient advocacy organizations’ selected from Gale’s Encyclopedia of Associations: National Organizations of the U.S. - **Focus:** industry (‘any for-profit company from any sector’, including pharmaceutical, device, and/or biotechnology) - **Country:** U.S. | - **Reported funding, total revenue:** Mailed survey (2013 – 2014) | - Receiving funding - Reporting on funding amount - Funding amount - Proportion of total revenue attributable to funding source - Policy for dealing with industry - COI concerns | ---- | Edmond J. Safra Center for Ethics at Harvard University | 1/4 paid member of a data monitoring committee for Genzyme Sanofi |
| McCoy 2017[^12^](#_ENREF_12) | Quantitative | - **Organizations:** 104 largest U.S.-based ‘patient-advocacy organizations’^[[15]](#footnote-15)^ - **Focus:** drug, device, or biotechnology industry - **Country:** U.S. | - **Reported funding, employment of board members’:** survey of websites, annual reports (2016) - **Total revenue:** survey of federal tax Forms 990 (2016) | - Receiving funding - Reporting on funding amount - Funding amount - Proportion of annual revenue attributable to funding source - Reporting on direction of use of funding - Policy for dealing with industry | - Employment of board members by drug, device, or biotechnology company | Not reported | 1/6 – personal fees 1/6 - spouse employment by a patient advocacy organization |
| Hemminki 2010[^13^](#_ENREF_13) | Mixed methods | - **Organizations:** 55 organizations from lists available from RAY^[[16]](#footnote-16)^ and the joint body of patient organizations and 13 ‘patient organizations’ selected through purposive sampling^[[17]](#footnote-17)^ - **Focus:** pharmaceutical industry - **Country:** Finland | - **Reported funding:** e-mail survey (2003) (n=55) - **Perspective of cooperation with the industry:** interviews (2004) (n=13), survey of websites (n=13) | - Receiving funding - Reporting on funding amount - Funding amount - Proportion of annual budget attributable to funding source - Policy for dealing with industry - Perspectives of stakeholders - Justification for patient organizations’ cooperation with the industry | ---- | Ministry of Education (The National Post Graduate School in Social and Health Policy, Management and Economics; and Doctoral Programmes in Public Health) and STAKES (now THL) | None |
| García-Sempere 2005[^14^](#_ENREF_14) | Quantitative | - **Organizations:** Key informants from 21 ‘Patient organizations’ in Spain - **Focus:** pharmaceutical industry, other health industries - **Country:** Spain | - **Reported funding:** semi-structured e-mail questionnaire (2003 – 2004) | - Receiving funding - Policy for dealing with industry | ---- | Agency of Quality of the Ministry of Health and Consumption (Spain) | Not reported |
| Jørgensen 2004[^15^](#_ENREF_15) | Quantitative | - **Organizations:** 13 ‘advocacy groups’ and 3 ‘consumer organizations’ - **Focus:** industry - **Country:** international (Scandinavian and English speaking countries^[[18]](#footnote-18)^) | - **Reported funding:** survey of websites^[[19]](#footnote-19)^ (2002); contact of organizations | - Receiving funding - Direction of use of funding - Policy for dealing with industry - Effect of funding (association between funding and information presented on the organizations’ websites) | ---- | Not funded | 1/2 involved in a systematic review that questioned the value of breast cancer screening |
| Colombo 2012[^16^](#_ENREF_16) | Quantitative | - **Organizations:** 157 Italian ‘Patient and consumer groups’ cited on websites of selected drug companies^[[20]](#footnote-20)^ - **Focus:** pharmaceutical industry - **Country:** Italy | - **Disclosed funding by organizations:** survey of websites and financial reports (website identification and assessment: 2010); contact of organizations that did not publicly share a COI policy | - Acknowledging funding - Reporting on funding amount - Reporting on proportion of annual income attributable to funding source - Reporting on direction of use of funding - Direction of use of funding (including unrestricted funding) - Number of funders - Policy for dealing with industry | ---- | “No external funding was received for this study” | “Grant from Pfizer” |
| Rothman 2011[^17^](#_ENREF_17) | Quantitative | - **Organizations:** 161 ‘Health Advocacy Organizations’ (U.S.) listed on the Lilly Grant Registry (2007) - **Focus:** pharmaceutical industry (Eli Lilly and Company) - **Country:** U.S. | - **Disclosed funding:** Survey of websites, annual reports and federal tax Forms 990 (2008 – 2009^[[21]](#footnote-21)^) - **Net sales and therapeutic areas of Eli Lilly and Company:** Review of 2007 annual report of Eli Lilly | - Acknowledging funding - Reporting on funding amount - Alignment between funding and industry’s interests | ---- | The May and Samuel Rudin Family Foundation, the Pew Charitable Trusts, and the Institute on Medicine as a Profession | 2/4 - consultants to the Office of the Attorney General of the State of Texas in litigation against Johnson & Johnson related to Risperidone |
| Lau 2018[^18^](#_ENREF_18) | Quantitative | - **Organizations:** Random sample of 133 ‘health consumer organizations’ with a website listed in Medicines Australia reports from 2013 to 2016^[[22]](#footnote-22)^ - **Focus:** pharmaceutical industry - **Country:** Australia | - **Disclosed funding, employment of board members:** survey of websites and annual reports (data collection date: 2017; annual reports date: 2013 - 2016) - **Total funding amount:** survey of Medicines Australia reports | - Acknowledging funding - Reporting on funding amount - Reporting on proportion of total income attributable to funding source - Reporting on direction of use of funding - Direction of use of funding (including unrestricted) - Reporting on identity of funders - Policy for dealing with industry | - Employment of board members by pharmaceutical companies | Not reported | 1/3 - Expert witness on behalf of plaintiffs in a Canadian class action suit concerning cardiovascular risks of a testosterone gel |
| Jones 2008[^19^](#_ENREF_19) | Mixed methods | - **Organizations:**   - Study 1: phase 1: 123 ‘consumer groups’; phase 2: 39 leaders from a sample of ‘consumer groups’; phase 3: 31 representatives from the health professions, government, Parliament, charities and ABPI   - Study 2: 4 Representatives (senior officers) from pharmaceutical companies and ABPI, 3 government, and 3 industry observers, including a consumer group   - Study 3: 246 ‘consumer groups’ identified by the ABPI’s^[[23]](#footnote-23)^ full-members - **Focus:** pharmaceutical industry - **Country:** UK | - **Views on the relationship between organizations and the industry**    - Study 1: phase 1: semi-structured postal questionnaire ; phase 2: semi-structured interviews; phase 3: semi-structured interviews) (1999 – 2001)   - Study 2: Semi-structured interviews (2005 – 2006) - **Disclosed funding (study 3):** Survey of websites (May, Nov 2007) | - Acknowledging funding - Reporting on funding amount - Reporting on proportion of total income attributable to funding source - Reporting on direction of use of funding - Direction of use of funding (including unrestricted) - Reporting on identity of funders - Policy for dealing with industry - Perspectives of stakeholders - Categorization of the relationship between organizations and the industry - Justification for patient organizations’ cooperation with the industry | ---- | Economic and Social Research Council | Not reported |
| Leto di Priolo 2012[^20^](#_ENREF_20) | Quantitative | - **Population:** 46 Policy makers, 54 cancer healthcare group representatives, and 61 cancer ‘patient group’ leaders from a convenience sample of 12 EU countries^[[24]](#footnote-24)^ - **Focus:** pharmaceutical industry - **Country:** 12 EU countries^x^ | **Opinion on the relation between ‘patient groups’ and pharmaceutical companies:** telephone interview using a structured questionnaire (2009) | - Perspectives of stakeholders - Justification for patient organizations’ cooperation with the industry | ---- | Novartis Oncology Region Europe | 4/4 - financial relationships with industry |
| Wranik 2019[^21^](#_ENREF_21) | Qualitative | - **Population:** 18 members of the Transparency Council in Poland, and 11 members of pERC^[[25]](#footnote-25)^ or similar sub-national cancer committees in Canada - **Focus:** pharmaceutical industry - **Country:** Poland and Canada | **‘Threats’ to HTA^[[26]](#footnote-26)^ process:** qualitative interviews in person in Poland, and via telephone in Canada (2017 – 2018) | - Perspectives of stakeholders | ---- | European Union Horizon 2020 research and innovation programme and Canadian Institutes for Health Research Partnerships for Health Systems Improvement | "No financial COI", one author is affiliated with the pan Canadian Oncology Drug Review |

**References**

1. O'Donovan O. Corporate colonization of health activism? Irish health advocacy organizations' modes of engagement with pharmaceutical corporations. *International Journal of Health Services* 2007;37(4):711-33.

2. Pinto D, Martin D, Chenhall R. The involvement of patient organisations in rare disease research: a mixed methods study in Australia. *Orphanet journal of rare diseases* 2016;11(1):2.

3. Pinto D, Martin D, Chenhall R. Chasing cures: Rewards and risks for rare disease patient organisations involved in research. *BioSocieties* 2017;13(1):123-47.

4. Li DG, Singer S, Mostaghimi A. Prevalence and Disclosure of Potential Conflicts of Interest in Dermatology Patient Advocacy Organizations. *JAMA dermatology* 2019

5. Lexchin J. Association between commercial funding of Canadian patient groups and their views about funding of medicines: An observational study. *PloS one* 2019;14(2):e0212399.

6. Fabbri A, Swandari S, Lau E, et al. Pharmaceutical Industry Funding of Health Consumer Groups in Australia: A Cross-sectional Analysis. *International Journal of Health Services* 2019:0020731418823376.

7. Mandeville KL, Barker R, Packham A, et al. Financial interests of patient organisations contributing to technology assessment at England’s National Institute for Health and Care Excellence: policy review. *Bmj* 2019;364:k5300.

8. Peterlein C, Friedrich S, Daniel H, et al. Evaluation of Organisational Structures of Self-help Groups in the Field of Paediatric Orthopaedics. *Zeitschrift fur Orthopadie und Unfallchirurgie* 2018

9. Industry funding of cancer patient advocacy organizations. Mayo Clinic Proceedings; 2016. Mayo Foundation for Medical Education and Research.

10. Ball DE, Tisocki K, Herxheimer A. Advertising and disclosure of funding on patient organisation websites: a cross-sectional survey. *BMC Public Health* 2006;6:201.

11. Rose SL, Highland J, Karafa MT, et al. Patient Advocacy Organizations, Industry Funding, and Conflicts of Interest. *JAMA Internal Medicine* 2017;177(3):344-50. doi: <https://dx.doi.org/10.1001/jamainternmed.2016.8443>

12. McCoy MS, Carniol M, Chockley K, et al. Conflicts of Interest for Patient-Advocacy Organizations. *New England Journal of Medicine* 2017;376(9):880-85. doi: <https://dx.doi.org/10.1056/NEJMsr1610625>

13. Hemminki E, Toiviainen HK, Vuorenkoski L. Co-operation between patient organisations and the drug industry in Finland. *Soc Sci Med* 2010;70(8):1171-5. doi: <https://dx.doi.org/10.1016/j.socscimed.2010.01.005>

14. Garcia-Sempere A, Artells JJ. [Organization, functioning and expectations of organizations representing patients. Survey of key informants]. *Gac Sanit* 2005;19(2):120-6.

15. Jørgensen KJ, Gøtzsche PC. Presentation on websites of possible benefits and harms from screening for breast cancer: cross sectional study. *Bmj* 2004;328(7432):148.

16. Colombo C, Mosconi P, Villani W, et al. Patient organizations’ funding from pharmaceutical companies: is disclosure clear, complete and accessible to the public? An Italian survey. *PLoS One* 2012;7(5):e34974.

17. Rothman SM, Raveis VH, Friedman A, et al. Health advocacy organizations and the pharmaceutical industry: an analysis of disclosure practices. *American Journal of Public Health* 2011;101(4):602-9. doi: <https://dx.doi.org/10.2105/AJPH.2010.300027>

18. Lau E, Fabbri A, Mintzes B. How do health consumer organisations in Australia manage pharmaceutical industry sponsorship? A cross-sectional study. *Aust Health Rev* 2018;19:19. doi: <https://dx.doi.org/10.1071/AH17288>

19. Jones K. In whose interest? Relationships between health consumer groups and the pharmaceutical industry in the UK. *Sociol Health Illn* 2008;30(6):929-43. doi: <https://dx.doi.org/10.1111/j.1467-9566.2008.01109.x>

20. di Priolo SL, Fehervary A, Riggins P, et al. Assessing stakeholder opinion on relations between cancer patient groups and pharmaceutical companies in Europe. *The Patient-Patient-Centered Outcomes Research* 2012;5(2):127-39.

21. Wranik WD, Zielińska DA, Gambold L, et al. Threats to the value of Health Technology Assessment: Qualitative evidence from Canada and Poland. *Health Policy* 2019;123(2):191-202.

1. Published as mixed methods and the outcomes of interest were obtainef from the quantitative part only [↑](#footnote-ref-1)
2. Rare Voices Australia and the Association of Genetic Support of Australasia (now Genetic Alliance Australia) [↑](#footnote-ref-2)
3. Common Drug Review (CDR); pan-Canadian Oncology Drug Review (pCODR) [↑](#footnote-ref-3)
4. In 2015-2018 on three projects: one looking at indication-based prescribing (United States Agency for Healthcare Research and Quality), a second to develop principles for conservative diagnosis (Gordon and Betty Moore Foundation) and a third deciding what drugs should be provided free of charge by general practitioners (Government of Canada, Ontario Supporting Patient Oriented Research Support Unit and the St Michael’s Hospital Foundation) [↑](#footnote-ref-4)
5. Panel that discussed a pharmacare plan for Canada (Canadian Institute, a for-profit organization) [↑](#footnote-ref-5)
6. Canadian Institutes of Health Research [↑](#footnote-ref-6)
7. National Health and Medical Research Council [↑](#footnote-ref-7)
8. Health Action International [↑](#footnote-ref-8)
9. Pharmaceutical Benefits Scheme (PBS) [↑](#footnote-ref-9)
10. Pharmaceutical Benefits Advisory Committee (PBAC) [↑](#footnote-ref-10)
11. National Institute for Health and Care Excellence [↑](#footnote-ref-11)
12. Association of British Pharmaceutical Industry [↑](#footnote-ref-12)
13. National Comprehensive Cancer Network [↑](#footnote-ref-13)
14. Cancer, heart disease, diabetes, asthma, cystic fibrosis, epilepsy, depression, Parkinson's disease, osteoporosis, rheumatoid arthritis [↑](#footnote-ref-14)
15. Annual revenues ≥ USD $7.5 million [↑](#footnote-ref-15)
16. Finland's Slot Machine Association [↑](#footnote-ref-16)
17. Sampling represents old and new organizations, large and small organizations, and reflects different health conditions [↑](#footnote-ref-17)
18. Australia, Canada, Denmark, New Zealand Norway, Sweden, the United Kingdom, and the United States [↑](#footnote-ref-18)
19. Authors also contacted the organizations when information on funding was unclear [↑](#footnote-ref-19)
20. Among top 15 global corporations for sales in 2009, in addition to a group of Italian companies important for the Italian market [↑](#footnote-ref-20)
21. Date of annual reports and federal tax Forms 990: 2007 [↑](#footnote-ref-21)
22. Identified through Health Consumer Organization Support Reports provided by Australian pharmaceutical companies [↑](#footnote-ref-22)
23. Association of British Pharmaceutical Industry [↑](#footnote-ref-23)
24. Pre-selected to include a mix of small and large, new and old EU members (France, Germany, Hungary, Italy, Latvia, the Netherlands, Poland, Portugal, Romania, Spain, Sweden, and the UK) [↑](#footnote-ref-24)
25. pan Canadian Oncology Drug Review Expert Review Committee [↑](#footnote-ref-25)
26. Health technology assessment [↑](#footnote-ref-26)
