# Supplementary material for: Financial relationships between patient and consumer representatives and the health industry: A systematic review
Source: Health Expect. 2019 Dec 19;23(2):483–95. doi: 10.1111/hex.13013 (PMC7104632; doi:10.1111/hex.13013)
Supplement: Supplementary file 5 [file HEX-23-483-s005.docx]

**Appendix 5a: Results of studies addressing receiving funding among patients’ organizations^[[1]](#footnote-1)^**

| Author Year | Receiving industry funding | Funding amount | Proportional financial contribution | Policy for dealing with the industry | Other outcomes |
| --- | --- | --- | --- | --- | --- |
| O’Donovan 2007[^1^](#_ENREF_1) | 47%* | Not reported | Not reported | Do not accept pharmaceutical industry sponsorship: ‘a small number of organizations’ | Categorization of the relationship between organizations and the industry: corporatist, cautious cooperation, confrontational  Justification for patient organizations’ cooperation with the industry: common interests (e.g. development of new therapies, drug approval), insufficient public funding, no other choice |
| Pinto 2016[^2^](#_ENREF_2), Pinto 2017[^3^](#_ENREF_3) | 20%* (12/61) | Not reported | Not reported | Not reported | Not reported |
| Li 2019[^4^](#_ENREF_4) | 63% (15/24) (based on annual report)  42% (10/24) (based on website)   - 71%* (17/24) (based on annual report or website) - Remaining: no further data provided by the authors | - Not reported: 50%* (12/24) - Out of those who reported (n=12):   - ≥$1,000,000: 25%†* (3/12)   - $500,000-999,999: 8%† (1/12)   - $100,000-499,999: 33%† (4/12)   - <$100,000: 33%† (4/12) | Calculated industry sponsorship contribution to total donations:   - ≥40%: 8% (2/24) - 10%-39.9%: 8% (2/24) - <10%: 33% (8/24) - Could not be determined: 50% (12/24) | Not reported | Reporting on direction of use of funding: 0%*  Reporting on identity of funders:  Disclosed the names of individual donors:   - 58% (14/24) (based on annual report) - 42% (10/24) (based on website) - 67% (16/24) (based on annual report or website)   Board members with COI^[[2]](#footnote-2)^:  Current/former industry executive on the governing board:   - 13%* (3/24) (based on annual report/website) - 54%* (13/24) (based on LinkedIn search) |
| Lexchin 2019[^5^](#_ENREF_5) | - Funding declared: 87.1%* (324/372) of submissions   - Funding by the company marketing the product: 86.1% (279/324)   - Remaining: funding by other companies - No funding declared: 8.06%† (30/372) - Funding could not be determined: 4.03%† (15/372) | - Ranges^[[3]](#footnote-3)^ (21 submissions):   $0-$5,000: 18 companies  $5,000 - $10,000: 12 companies  $10,001 - $50,000: 20 companies  >$50,000: 43 companies | - - Submissions stating proportion of annual budget attributable to donations: ‘20 submissions (8 different patient groups)’ out of 324 submissions in which groups declared funding   - ‘Many of the 8 groups were inconsistent in providing that information’   - Range: <2% - 36% | Not reported | Median number of conflicts per submission:  CDR = 7 (IQR 4-10); pCODR = 1 (IQR 1-5)  Reporting on the direction of use of funding:  81.7% of declarations naming the donor companies did not specify how the received money was used (‘occasionally’ groups gave vague statements about how the money was used, e.g., for research or educational events)  Effect of funding^[[4]](#footnote-4)^:   - No association between funding and recommendations (*p*-value=0.3117) - No association between funding and difference of recommendations between patient groups, and CDR and pCODR (*p*-value = 0.78) |
| Fabbri 2019[^6^](#_ENREF_6) | Not applicable | Amount spent per sponsorship:   - Mean: AU$23,206.33 (SD $46,971.72) - Median: AU $10,000 (IQR $3,000–25,000) - <AU $20,000: 63.9% of sponsorships - > AU$100,000: 2.8% of sponsorships | Not reported | Not reported | Number of sponsorships per organization:  Mean = 6.5 (SD 9.51)  Median = 3 (IQR 1–8)  Direction of use of funding:  Information/information materials: 50.9%; program and project: 35.2%; disease awareness: 43.9%; education: 49.1%; meetings: 53.9%; research: 28.3%; advocacy: 22.2%; fundraising event: 37%; unrestricted: 10%*; exposition: 6.5%; travel: 15.7%; guideline: 4.3%; miscellaneous: 16.5%  Alignment between funding and industry’s interests:  Main funders^[[5]](#footnote-5)^ of the top 5 consumer groups (receiving most funding) were companies that manufactured drugs for conditions covered by these groups and that were under review for PBS^[[6]](#footnote-6)^ listing |
| Mandeville 2019[^7^](#_ENREF_7) | - 72%*   - By manufacturer(s) of technology under appraisal: 64%   - By manufacturer(s) of competitor products: 64%) - Funding known to NICE’s ^[[7]](#footnote-7)^ committees out of all declarable funding: 41% - Funding unknown to NICE’s7committees owing to NICE’s7 disclosure policy out of all unknown funding: 62% - Funding known to NICE’s7 committees out of all funding identified: 21% | - Median=0; IQR = £0-£25,750 (based on accounts, annual reports, or websites) - Median=£283; IQR = £0-£5,120 (based on ‘Disclosure UK’) - Median=£37,707; IQR = £2,500-£135,081 (based on individual manufacturers’ disclosures) - Median=£38,754; IQR=£6,375-£105,485 (based on all sources combined) | Calculated pharmaceutical industry funding contribution to income:   - Range= <1% - 70% - Median=0%; IQR=0-1 (based on accounts, annual reports, or websites) - Median=0.01%; IQR=0-0.3 (based on ‘Disclosure UK’) - Median=1.9%; IQR=0.3-12.5 (based on individual manufacturers’ disclosures) - Median=3.1%; IQR=0.1-24.6 (based on all sources combined) | Have policies on pharmaceutical industry funding: 30%* (16/53)   - Do not accept funding: 13%† (2/16) out of organizations with a policy, or 3%†* out of all organizations (2/53) - Committed to disclosing any funding in annual reports or websites: 75%† (12/16) - Conditions on funding^[[8]](#footnote-8)^: 69%† (11/16) - Specific clauses for policy and health technology assessment activities: 18%† (3/16) | Number of funders:   - Median=0; IQR=0-2 (based on accounts, annual reports, or websites) - Median=0.5; IQR=0-1 (based on ‘Disclosure UK’) - Median=1.5; IQR=0.5-2.7 (based on individual manufacturers’ disclosures) - Median=2; IQR=0.5-5.5 (based on all sources combined) |
| Peterlein 2018[^8^](#_ENREF_8) | Did not receive any donations: >80% | Not reported | Not reported | Not reported | Not reported |
| Abola 2016[^9^](#_ENREF_9) | - Reported biopharmaceutical sponsors: 75%* (51/68) - Did not report: 23.5% (16/68) | Not reported | Not reported | Does not accept money from the industry: 1.5%* (1/68) | Number of funders:  Median = 7; range = 1-19 |
| Ball 2006[^10^](#_ENREF_10) | - 45%* (31/69) - Remaining: 40.5%† (28/69) gave no indication; when contacted, 44%† (4/9) of those who responded affirmed receiving funding - Pharma-sponsored events or programs: 17% (12/69) | Specified: 0%* | Specified: 0%* | Not reported | Direction of use of funding  Gave some indication of what the funding was used for (core operations, education or research): 16%* (11/69); unrestricted funding: 13%* (9/69) (‘although implied in some additional cases’)  Number of funders:   - Median = 6; range = 0-38 (based on annual reports) - Median = 1; range = 0-21 (based on websites^[[9]](#footnote-9)^) |
| Rose 2017[^11^](#_ENREF_11) | 67.3%* (165/245)  Remaining: no further data provided by the authors | Provided information on their funding from for-profit companies: 240/289*   - Median=$15,000; IQR=$0-$102,500 - Ranges   ≤ $9,999: 43.8% (105/240)  ≥ $1 million: 8.8%* (21/240)  Among the subset that reported receiving industry funding:   - Median=$50,000; IQR=$15,000-$200,000 | - Industry contribution to total funding (last fiscal year):   >25%: 33.8% (54/160)  >50%: 11.9% (19/160)   - Pharmaceutical, device, and/or biotechnology companies contribution to industry support (last 3 years)^[[10]](#footnote-10)^:   Median=45%; IQR=0%-100% | - Written organizational COI policy: 63.9%* - Written policies ‘are very good at addressing key components of COI’: 55.0% | COI concerns:   - Organization perceived pressure to conform its positions to the interests of corporate donors or partners: 7.7% - Organization had declined a contribution because of concerns about COI: 13.7% - COI are very or moderately relevant to PAOs: 81.8% |
| McCoy 2017[^12^](#_ENREF_12) | 83%* (86/104)  Remaining (n=18): 13 provided no donor information; 1 explicitly mentioned it does not accept industry support | - Published the amounts of donation: 57%† (59/104) - Specified the total amount received from industry or corporate donations: 17%* (18/104) - Annual revenue from drug, device, or biotechnology industry donations^[[11]](#footnote-11)^:   ≥ $1 million: 39%* (23/59)  < $1 million: 22% (13/59)  Could not be determined: 39% (23/59) | Calculated industry donations contribution to annual income1^1^:   - ≥ 10%: 19% (11/59) - < 10%: 34% (20/59) - Could not be determined: 47% (28/59) | - Explicitly indicating it does not accept drug, device, or biotechnology industry support: 1%†* (1/104) - Published any policy pertaining to COI: 26%†* (27/104) - Policy that addressed institutional COI : 11.5%† (12/104) | Reporting on the direction of use of funding:  9.6%* (10/104) provided information about how individual donations were used  Board members with COI^[[12]](#footnote-12)^:   - Current drug, device, or biotechnology industry executive:   ≥ 1 on the governing board: 36% (37/104); ≥ 1 in a leadership position on the board (such as chair or vice-chair): 12% (12/104)   - Former drug, device, or biotechnology industry executive:   ≥ 1 on the governing board: 4%† (4/104); ≥ 1 in a leadership position: 1%† (1/104)   - Current or former drug, device, or biotechnology industry executive:   ≥ 1 on the governing board: 40%†* (41/104); ≥ 1 in a leadership position on the board (such as chair or vice-chair): 13†% (13/104) |
| Hemminki 2010[^13^](#_ENREF_13) | - Any financial support^[[13]](#footnote-13)^: 71%* (39/55) - Money donations: 11% | - Exact amount of support available: 38%†* (21/55) organizations - Range = €300 –€58,000 - ‘None of the patient organizations refused reporting the amount of support, but the question was sometimes left unanswered or vaguely answered’ | Drug industry contribution to annual budget:  >20%: 20% (4/20) | Interviews:  Own ethical codes for cooperation with industry (current or in development): 3/13 | Justification for patient organizations’ cooperation with the industry: facilitating communication and transmittal of information, common interests (better reimbursement of drugs), learning about good marketing skills  Perspectives of stakeholders: Concerns related to co-operation between organizations and industry (stainability, conditions imposed by the industry, and power balance); reporting of threat to independence and objectivity and becoming stigmatized as ‘‘a drug firm marketing tool’’ as a problem in cooperation with drug firms by 27.5%^[[14]](#footnote-14)^† of organizations (11/40) |
| García-Sempere 2005[^14^](#_ENREF_14) | Health industry: 61.9%†*   - Pharmaceutical industry: 47.6% (10/21) - Other health industries: 14.3% (3/21) | Not reported | Not reported | Code that regulates financial relationships: 52.4%* (11/21) | Not reported |
| Jørgensen 2004[^15^](#_ENREF_15) | - 87.5%†* (14/16)   - 100%† (13/13) advocacy groups accepted sponsorship from industry   - 67%† (2/3) consumer groups disclosed not accepting grants from industry | Not reported | Not reported | Do not accept grants from industry: 12.5%†* (2/16) | Direction of use of funding:  1/16 group noted that funding is restricted to general operating support, 13/16 groups accepted sponsorship from industry, apparently without restrictions  Effect of funding   - Information presented on websites of organizations that accepted financial support from industry was ‘selective and biased and failed to mention major harms’ - Organizations which questioned the value of screening did not have an apparent COI |
| di Priolo 2012[^16^](#_ENREF_16) | Not applicable | Not applicable | Not applicable | Not applicable | Perspectives of stakeholders:   - 59%† had an overall very positive/somewhat positive opinion of the relationship between patient groups and the industry– main reason was because patient groups benefit from industry’s resources (e.g. funding, information, advice); 4% had a very negative opinion - Respondents favored governments, and charitable foundations to the pharmaceutical industry as a funding source - Most positive funding approach was project funding from multiple companies (60%); negative funding approaches included funding from a single company (19%) and core funding (24%) - 42% were very dissatisfied/somewhat dissatisfied with the ‘current controls and safeguards’ on the relationship - Concerns raised about the relationship being unequal, conflicts between the industry’s interests and advocacy interests, motives behind the funding, and the risks to patients’ organizations’ credibility   Justification for patient organizations’ cooperation with the industry: common interests (‘developing safe and effective treatments’, ‘ensuring fair, accurate, and non-promotional information is available to patients’, ‘prevention and screening programs’, ‘maintaining credibility and trust with other stakeholders’, ‘promoting medicines adherence’, ‘developing tools to communicate with patients’, ‘ensuring patient access to new treatments  as quickly as possible’, ‘policy making’, ‘pharmacovigilance’) |
| Wranik 2019[^17^](#_ENREF_17) | Not applicable | Not applicable | Not applicable | Not applicable | Perspectives of stakeholders:  Funding of patients’ organizations seen as:   - Internal threat to HTA^[[15]](#footnote-15)^: when members of patients’ organizations serve on committees - External threat to HTA^15^: incorporation of potentially biased information obtained from patients’ organizations |

**Appendix 5b: Results of studies addressing acknowledgement of funding of patients’ organizations**

| *Author, Year* | *Acknowledging funding* | *Funding amount* | *Proportional financial contribution* | *Policy for dealing with the industry* | *Other outcomes* |
| --- | --- | --- | --- | --- | --- |
| Pinto 2016[^2^](#_ENREF_2), Pinto 2017[^3^](#_ENREF_3) | - 36%†* (4/11)^[[16]](#footnote-16)^ | Not reported | Not reported | Not reported | Not reported |
| Colombo 2012[^18^](#_ENREF_18) | - Named at least 1 pharmaceutical company as providing funding: 29%* (46/157)   - Stated at least one drug company corresponding to the disclosure made by the drug company itself: 65% (30/46)   - Declared other drug companies as sponsors: 35% (16/46)   - 25%* of the sponsorships disclosed corresponded to the disclosure made by the drug companies considered | Reported the amount received: 6%* (n=3/46) | Reported on drug industry contribution to income: 0%* | - Published codes of conduct dealing with sponsorship: 9%* (14/157) - 84% of those without websites contacted (n=85); 3 (4%) responded:   - Had no code of conduct about sponsors: n=2   - Had a code but did not specify the norms or give examples of its application: n=1 | Number of funders  Range: 1–11  Direction of use of funding:   - Named activities funded: 54%* (25/46) - Core activities: 40% (10/25) - Educational activities: 88% (22/25) - Research activities: 28% (7/25) - Stated that funding was unrestricted: 19.5%†* (9/46) |
| Rothman 2011[^19^](#_ENREF_19) | - 25%* (40/161) (anywhere on the website) - 18% (29/161) (based on annual report) - 1% (2/161) (based on corporate sponsors page) - 10% (16/161) (declared funder as the sponsor of a grant event) | Reported the amount received: 0.6%* (1/161) | Not reported | Not reported | Alignment between funding and industry’s interests:  HAOs active in Lilly’s 3 main therapeutic areas received 94% of Lilly’s grants to HAOs |
| Lau 2018[^20^](#_ENREF_20) | Acknowledged receiving funding: 52.3%* (68/130)  Remaining (62/130):   - Corporate funding reported, not specific to pharmaceutical industry: 22.3% (29/130) - Funding or donation reported, not specific to corporate funding: 10.0% (13/130) - No financial information publicly available: 15.4% (20/130)   Compared to Medicines Australia reports:   - 23.9%* (16/67) out of those acknowledging reported exactly the same donors - 23.9% (16/67) did not report at least one donor - 31.3% (21/67) reported donors that were Medicines Australia members but were not listed in the reports - 20.9% (14/67) missed donors and reported extra donors | Provided information on the amount of industry funding:   - In total: 13.2%* (9/68) - Per donor: 10.4% (7/67) | Reported on industry contribution to income: 4.4%* (3/68) | - Corporate sponsorship policies publicly available on their websites: 18%* (24/133) - 67%† (16/24) had policies concerning organizational independence - 63%† (15/24) had provisions related to disclosure or management of conflicts of interest of members, including the board - 58%† (14/24) addressed relationships with corporate sponsors - 50%† (12/24) mentioned financial transparency - 38%† (9/24) had policies specific to pharmaceutical industry funding (vs corporate in general) - 25%† (6/24) regulated advertising from sponsors) - Policies but the content was not publicly available: 1.5% (2/133) | Direction of use of funding:  Reported on the direction of use of funding (activities, projects, unrestricted): 52.9%* (36/68)  Identity of funders:   - Stated the identity of donors: 98.5% (67/68) - Discrepancies when compared to Medicines Australia reports (refer to column ‘acknowledging funding’)   Board members with COI^[[17]](#footnote-17)^:   - Current or previous employees of pharmaceutical companies in the governing board: 70.9% (83/117) out of organizations providing information, or 64%†* (83/130) out of total organizations - Current or previous employees of pharmaceutical companies in the advisory board: 10.5% (2/19) |
| Jones 2008[^21^](#_ENREF_21) | - No website: 10% (25/246) - ‘Nearly two-thirds gave no online financial information, although some provided access to annual reports which may acknowledge links’ - Acknowledging funding: 26% (64/246) out of total organizations, or 29% (64/221)†* out of groups with a website | Listed the amount of funding received: 22.7%†* (5/22) of groups receiving grants from ≥5 companies | Reported on industry contribution to income: 6%†* (4/64) | - Policies for working with industry: 1.8%†* (4/221) - Gave details of the policy governing relationships: 36.4%† (8/22) of groups receiving grants from ≥5 companies   Interviews:   - Policy guidelines: 9/34 - Refuse contact with industry: 2/34 | Direction of use of funding:  Gave details of the type of grant provided (mainly educational or unrestricted) and their purpose (e.g. sponsoring websites, funding publications): 28%†* (18/64)  Identity of funders:  Named individual companies: 34%† (22/64)  Perspectives of stakeholders: stakeholders stressed on the importance of disclosure and transparency; patient groups were concerned about industry links compromising their credibility, and about accusations of promoting drug makers’ products  Categorization of the relationship between organizations and the industry: organizations categorized as refusers, accepters, and non-disclosers  Justification for patient organizations’ cooperation with the industry: communication between ‘producer and consumer’, need for resources for provision of services that benefit patients, no source is conflict-free |

**References**

1. O'Donovan O. Corporate colonization of health activism? Irish health advocacy organizations' modes of engagement with pharmaceutical corporations. *International Journal of Health Services* 2007;37(4):711-33.

2. Pinto D, Martin D, Chenhall R. The involvement of patient organisations in rare disease research: a mixed methods study in Australia. *Orphanet journal of rare diseases* 2016;11(1):2.

3. Pinto D, Martin D, Chenhall R. Chasing cures: Rewards and risks for rare disease patient organisations involved in research. *BioSocieties* 2018;13(1):123-47.

4. Li DG, Singer S, Mostaghimi A. Prevalence and Disclosure of Potential Conflicts of Interest in Dermatology Patient Advocacy Organizations. *JAMA dermatology* 2019

5. Lexchin J. Association between commercial funding of Canadian patient groups and their views about funding of medicines: An observational study. *PloS one* 2019;14(2):e0212399.

6. Fabbri A, Swandari S, Lau E, et al. Pharmaceutical Industry Funding of Health Consumer Groups in Australia: A Cross-sectional Analysis. *International Journal of Health Services* 2019:0020731418823376.

7. Mandeville KL, Barker R, Packham A, et al. Financial interests of patient organisations contributing to technology assessment at England’s National Institute for Health and Care Excellence: policy review. *Bmj* 2019;364:k5300.

8. Peterlein C, Friedrich S, Daniel H, et al. Evaluation of Organisational Structures of Self-help Groups in the Field of Paediatric Orthopaedics. *Zeitschrift fur Orthopadie und Unfallchirurgie* 2018

9. Industry funding of cancer patient advocacy organizations. Mayo Clinic Proceedings; 2016. Mayo Foundation for Medical Education and Research.

10. Ball DE, Tisocki K, Herxheimer A. Advertising and disclosure of funding on patient organisation websites: a cross-sectional survey. *BMC Public Health* 2006;6:201.

11. Rose SL, Highland J, Karafa MT, et al. Patient Advocacy Organizations, Industry Funding, and Conflicts of Interest. *JAMA Internal Medicine* 2017;177(3):344-50. doi: <https://dx.doi.org/10.1001/jamainternmed.2016.8443>

12. McCoy MS, Carniol M, Chockley K, et al. Conflicts of Interest for Patient-Advocacy Organizations. *New England Journal of Medicine* 2017;376(9):880-85. doi: <https://dx.doi.org/10.1056/NEJMsr1610625>

13. Hemminki E, Toiviainen HK, Vuorenkoski L. Co-operation between patient organisations and the drug industry in Finland. *Soc Sci Med* 2010;70(8):1171-5. doi: <https://dx.doi.org/10.1016/j.socscimed.2010.01.005>

14. Garcia-Sempere A, Artells JJ. [Organization, functioning and expectations of organizations representing patients. Survey of key informants]. *Gac Sanit* 2005;19(2):120-6.

15. Jørgensen KJ, Gøtzsche PC. Presentation on websites of possible benefits and harms from screening for breast cancer: cross sectional study. *Bmj* 2004;328(7432):148.

16. di Priolo SL, Fehervary A, Riggins P, et al. Assessing stakeholder opinion on relations between cancer patient groups and pharmaceutical companies in Europe. *The Patient-Patient-Centered Outcomes Research* 2012;5(2):127-39.

17. Wranik WD, Zielińska DA, Gambold L, et al. Threats to the value of Health Technology Assessment: Qualitative evidence from Canada and Poland. *Health Policy* 2019;123(2):191-202.

18. Colombo C, Mosconi P, Villani W, et al. Patient organizations' funding from pharmaceutical companies: is disclosure clear, complete and accessible to the public? An Italian survey. *PLoS ONE* 2012;7(5):e34974. doi: <https://dx.doi.org/10.1371/journal.pone.0034974>

19. Rothman SM, Raveis VH, Friedman A, et al. Health advocacy organizations and the pharmaceutical industry: an analysis of disclosure practices. *American Journal of Public Health* 2011;101(4):602-9. doi: <https://dx.doi.org/10.2105/AJPH.2010.300027>

20. Lau E, Fabbri A, Mintzes B. How do health consumer organisations in Australia manage pharmaceutical industry sponsorship? A cross-sectional study. *Aust Health Rev* 2018;19:19. doi: <https://dx.doi.org/10.1071/AH17288>

21. Jones K. In whose interest? Relationships between health consumer groups and the pharmaceutical industry in the UK. *Sociol Health Illn* 2008;30(6):929-43. doi: <https://dx.doi.org/10.1111/j.1467-9566.2008.01109.x>

1. * Value used in summary statistics

   † Value calculated by the authors

   Percentages represent proportions out of total sample for each study, unless otherwise specified [↑](#footnote-ref-1)
2. Reported board member names on their website: 96% (23/24); provided board members’ employment information: 25% (6/24) [↑](#footnote-ref-2)
3. * Value used in summary statistics

   † Value calculated by the authors

   After September 1, 2017, submissions listed grants from companies in one of four brackets (some submissions named more than one company, some companies gave money to more than one group) [↑](#footnote-ref-3)
4. Excluding submissions involving subsequent entry biologics [↑](#footnote-ref-4)
5. Apart from 3 companies [↑](#footnote-ref-5)
6. Pharmaceutical Benefits Scheme (PBS) (government subsidization) [↑](#footnote-ref-6)
7. National Institute for Health and Care Excellence [↑](#footnote-ref-7)
8. E.g. a cap on the percentage of income received from industry or from one manufacturer [↑](#footnote-ref-8)
9. Only websites having an annual report for comparison [↑](#footnote-ref-9)
10. * Value used in summary statistics

    † Value calculated by the authors

    Groups who provided a detailed breakdown of their for-profit funding sources out of the groups that received industry funding (65%; 156/240) [↑](#footnote-ref-10)
11. Out of those specifying donation amounts (57%; 59/104) [↑](#footnote-ref-11)
12. Provided names of board members: 97% (101/104); provided board members’ employment information: 74% (77/104) [↑](#footnote-ref-12)
13. Includes advertising in magazines or newsletters, participation in organizing seminars, assistance in printing costs, participation in projects, giving money donations, arranging recreational services or travels, giving information on drugs, and getting expert opinions and lecturers [↑](#footnote-ref-13)
14. Out of organizations specifying problems related to co-operation with drug firms (40/55 organizations) [↑](#footnote-ref-14)
15. * Value used in summary statistics

    † Value calculated by the authors

    Health technology assessment [↑](#footnote-ref-15)
16. * Value used in summary statistics

    † Value calculated by the authors

    Based on websites, excluding linked documents (e.g., annual reports) [↑](#footnote-ref-16)
17. * Value used in summary statistics

    † Value calculated by the authors

    Provided information on board members on their websites (117/133); provided information on advisory board members on their websites (29/133) [↑](#footnote-ref-17)
